# Electrophysiological engineering of heart-derived cells with calcium-dependent potassium channels improves cell therapy efficacy for cardioprotection

**DOI:** 10.1038/s41467-021-25180-8

**Published:** 2021-08-16

**Authors:** Patrick Vigneault, Sandrine Parent, Pushpinder Kanda, Connor Michie, Darryl R. Davis, Stanley Nattel

**Affiliations:** 1 Research Center and Department of Medicine, Montreal Heart Institute, Université de Montréal, Montreal, QC Canada; 2 Division of Cardiology, Department of Medicine, University of Ottawa, Ottawa, ON Canada; 3 Department of Cellular and Molecular Medicine, Faculty of Medicine, University of Ottawa, Ottawa, ON Canada; 4 Department of Pharmacology and Therapeutics, McGill University, Montreal, QC Canada; 5 Institute of Pharmacology, West German Heart and Vascular Center, Faculty of Medicine, University Duisburg-Essen, Essen, Germany; 6 IHU LIRYC and Fondation Bordeaux Université, Bordeaux, France

**Keywords:** Ion channels, Cell therapies, Cardiology

## Abstract

We have shown that calcium-activated potassium (KCa)-channels regulate fundamental progenitor-cell functions, including proliferation, but their contribution to cell-therapy effectiveness is unknown. Here, we test the participation of KCa-channels in human heart explant-derived cell (EDC) physiology and therapeutic potential. TRAM34-sensitive KCa3.1-channels, encoded by the *KCNN4* gene, are exclusively expressed in therapeutically bioactive EDC subfractions and maintain a strongly polarized resting potential; whereas therapeutically inert EDCs lack KCa3.1 channels and exhibit depolarized resting potentials. Somatic gene transfer of *KCNN4* results in membrane hyperpolarization and increases intracellular [Ca^2+^], which boosts cell-proliferation and the production of pro-healing cytokines/nanoparticles. Intramyocardial injection of EDCs after *KCNN4-*gene overexpression markedly increases the salutary effects of EDCs on cardiac function, viable myocardium and peri-infarct neovascularization in a well-established murine model of ischemic cardiomyopathy. Thus, electrophysiological engineering provides a potentially valuable strategy to improve the therapeutic value of progenitor cells for cardioprotection and possibly other indications.

## Abstract (web summary)

Strategies to improve the function of damaged hearts with progenitor cells have stalled. Here, the authors show that gene transfer of a calcium-dependent potassium channel enhances the functional properties and ability of explant-derived cells to improve heart function after a heart attack.

## Introduction

Despite significant advances in the management of cardiac diseases, heart failure remains one of the top killers worldwide^1^. In response, stem cell therapy has emerged as a potential approach to prevent the progression of heart failure^2^ and, among the number of candidates proposed for cardiac cell therapy, heart explant-derived cells (EDCs) have been developed as a promising paracrine-based cell therapeutic^3,4^. Previous work has shown that EDCs are intrinsic cardiac CD105^+^ cells that reduce pathological cardiac remodeling and improve myocardial function^5^. Akin to noncardiac cell products, clinical comorbidities may attenuate the regenerative potency of EDCs^6–8^ but very few markers of product identity have been shown to clearly predict an inert cell product, which makes clinical reliability and translation challenging.

In this study, we focus on the regulation of membrane potential (V_mem_) and intracellular Ca^2+^, which can exert significant influence over stem cell properties^9^. We recently showed that the function of bone-marrow-derived mesenchymal stem cells and resident cardiac c-Kit^+^ cells is critically governed by the intermediate-conductance Ca^2+^-activated K^+^ channel KCa3.1 (encoded by the *KCNN4* gene)^10^. In both adult progenitor cell types, KCa3.1 channels open in response to store-operated Ca^2+^-entry (SOCE) to hyperpolarize the cell membrane, increase the driving force for Ca^2+^ entry, and enhance transmembrane Ca^2+^ flux. KCa3.1-channel inhibition decreased SOCE, which suggested that Ca^2+^ induced increases in KCa3.1 are necessary to optimize membrane potential during Ca^2+^ entry.

Based on these insights, we hypothesized that KCa3.1-channel activity might influence the behavior of therapeutically relevant cells, and that *KCNN4* overexpression might improve therapeutic efficacy by optimizing V_mem_ during SOCE. Here, we test this hypothesis and provide evidence that tailoring plasma-membrane ion-channel function influences the therapeutic efficacy of ex vivo expanded human heart cells using a promising adult cell therapeutic within an established immunodeficient mouse model of ischemic cardiomyopathy.

## Results

### Distinct endogenous currents exist in human EDCs

**Fig. 1:**
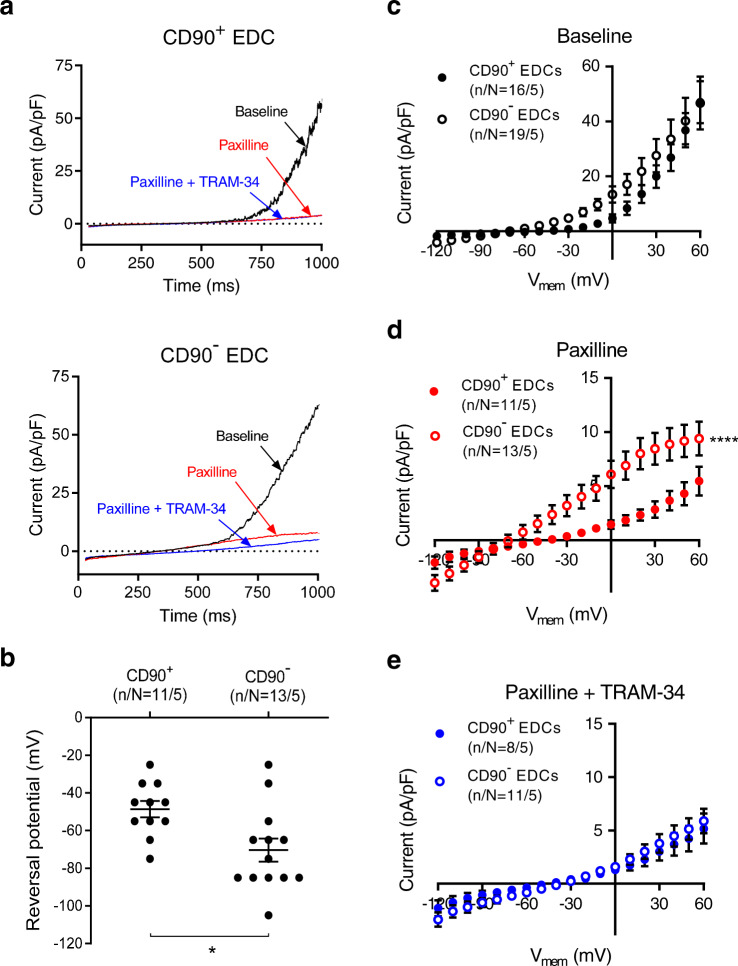
Endogenous ion currents expressed in human EDCs. **a** Original whole-cell currents from CD90^+^ (upper panel) and CD90^−^ EDCs (lower panel) under various experimental conditions, with a 1000-ms depolarizing voltage-ramp protocol from −120 to +60 mV, with a holding potential of −40 mV, before and after the addition of KCa inhibitors. Baseline = black, paxilline = red, paxilline + TRAM-34 = blue. **b** Reversal potential of whole-cell ion currents recorded in CD90^+^ and CD90^−^ EDCs in the presence of 300 nmol/L free-[Ca^2+^]_i_. **c**–**e** *I–V* relationship of whole-cell ion currents (recorded with 1000 ms voltage-ramps from −120 to +60 mV with a holding potential of −40 mV) in CD90^+^ and CD90^−^ EDCs under **c** basal conditions, **d** after addition of 1 µmol/L paxilline, or **e** after addition of 1 µmol/L paxilline and 1 µmol/L TRAM-34. Open circles = CD90^−^, closed circles = CD90^+^. Two-sided non-paired student *t*-test (panel **b**); two-way repeated-measures ANOVA showing significance of group effect; n/N = cells/cell lines per group. EDC explant-derived cell. Data in **b**–**e** are shown as individual data points along with mean and SEM. *P < 0.05; ****P < 0.0001 vs CD90^−^ EDCs. For precise *P* values, see Supplemental Source Data and Statistics file.

EDCs were obtained from patients with characteristics shown in Supplementary Table 1. Intriguingly, recent evidence has suggested that the major antigenic population found within ex vivo expanded heart-derived cells (the CD90 negative population, CD90^−^) constitutes the therapeutically active fraction within these heart-derived cell preparations and is responsible for most of the functional benefits associated with cell transplantation^11,12^. Thus, to characterize endogenous Ca^2+^ activated K^+^ (K_Ca_) channels within physiologically relevant subpopulations, CD90^+^ cells were fluorescently labeled before starting patch-clamp experiments. Under conditions that allow the activation of Ca^2+^-dependent current, we recorded outwardly rectifying voltage-dependent currents in both CD90^+^ and CD90^−^ cells (Fig. 1a). The reversal potential (E_rev_) of these currents averaged −51 ± 5 mV and −75 ±5 mV (*P* < 0.01) for CD90^+^ and CD90^−^ cells, respectively, (Fig. 1b). Although whole-cell ion-current density was found to be of the same order in both cell types (Fig. 1c), the differences in E_rev_ hinted that the two subpopulations possess distinct bioelectrical properties, and thus different profiles of plasma-membrane ion channels

### EDCs contain 2 types of Ca^2+^-dependent K^+^ currents with distinct contributions to V_mem_

**Fig. 2:**
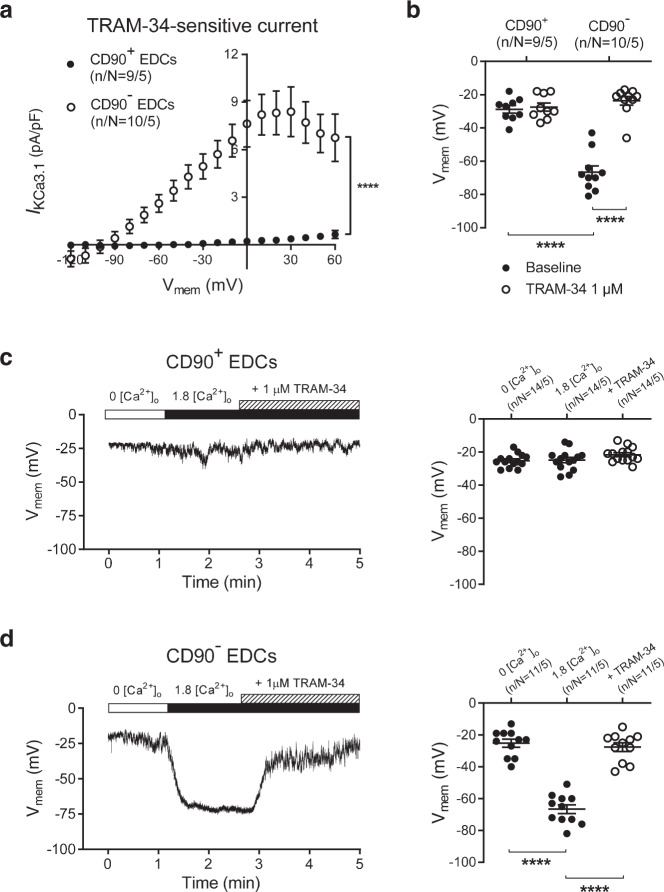
Functional endogenous KCa3.1 current and membrane-potential changes during SOCE in EDCs. **a**. *I–V* relationship of *I*_KCa3.1_ (TRAM-34–sensitive current) in CD90^+^ and CD90^−^ EDCs. Data are mean ± SEM; n/N = cells/cell lines per group. **b** Resting potential of EDCs before and after exposure to 1 µmol/L TRAM-34. Two-way repeated-measures ANOVA with individual-mean comparisons by Bonferroni-corrected two-sided *t*-tests; n/N = cells/cell lines per group. Data are individual points along with mean and SEM. **c** Original current-clamp recording from one CD90^+^ cell (left panel) and mean ± SEM data for V_mem_ changes induced by SOCE (resulting from intracellular Ca^2+^-depletion by exposure to nominally Ca^2+^-free extracellular Ca^2+^ (0 [Ca^2+^]_o_) and then reintroduction of 1.8 mmol/L Ca^2+^ (1.8 [Ca^2+^]_o_)), with subsequent exposure to 1-µmol/L TRAM-34 (right panel). **d** Original current-clamp recording from one CD90^−^ cell (left panel) and mean ± SEM data for V_mem_ changes induced by SOCE and subsequent exposure to 1 µmol/L TRAM-34 (right panel). One-way ANOVA with individual-mean comparisons by Bonferroni-corrected two-sided *t*-tests; numbers shown on bars are numbers of cells studied; n/N = cells/cell lines per group; Open circles = CD90^−^, closed circles = CD90^+^. EDC explant-derived cell, min minutes, V_me_ membrane potential. ****P < 0.0001. For precise *P* values, see Supplemental Source Data and Statistics file.

Since currents recorded in EDCs displayed some biophysical characteristics (like outward rectification) of the large-conductance Ca^2+^-dependent K^+^ current (*I*_BKCa_), we tested the response to the selective BK_Ca_-blocker paxilline. The outwardly rectifying current–voltage (*I–V*) relationship of EDCs was strongly affected by paxilline, leaving a substantial inwardly rectifying current in CD90^−^ cells and a smaller residual outwardly rectifying current in CD90^+^ cells (Fig. 1d). The paxilline-sensitive current representing *I*_BKCa_ was large and similar in both cell types (Supplementary Fig. 1a). V_mem_ (recorded with perforated-patch methods to maintain the physiological intracellular milieu) was significantly less negative in CD90^+^ cells versus CD90^−^ cells (−25 ± 2 mV vs. −64 ± 7 mV; *P* < 0.001), and was unaffected by paxilline (Supplementary Fig. 1b), suggesting that it is governed by another charge carrier(s). The differences in paxilline-resistant current between the 2 cell types was due to a TRAM-34-sensitive component, since their *I–V* relationships became indistinguishable after exposure to the selective KCa3.1-blocker TRAM-34 (Fig. 1e). In contrast to the substantial KCa3.1 currents (TRAM-34-sensitive; *I*_KCa3.1_) in the CD90^−^ subpopulation, *I*_KCa3.1_ was negligible in CD90^+^ cells (Fig. 2a). Blocking *I*_KCa3.1_ with TRAM-34 substantially depolarized V_mem_ of CD90^−^ EDCs without altering V_mem_ of CD90^+^ cells (Fig. 2b). In the presence of TRAM-34, the V_mem_ of CD90^−^ EDCs became similar to that of CD90^+^ cells. These results indicate that the V_mem_ of EDCs is largely determined by TRAM-34 sensitive K_Ca_3.1 conductance, which is substantial in CD90^−^ cells and undetectable in CD90^+^ cells.

### V_mem_ is controlled by Ca^2+^-dependent K^+^ currents during Ca^2+^-entry

One potential role for *I*_KCa3.1_ in EDCs would be hyperpolarization of V_mem_ to optimize Ca^2+^-inflow during SOCE, as previously demonstrated for cardiac-derived c-Kit^+^ cells and bone-marrow-derived mesenchymal stem cells^10^. To address this possibility, we induced SOCE in EDCs and monitored V_mem_ (Fig. 2c–d). In the presence of very low intracellular [Ca^2+^] resulting from perfusion with nominally Ca^2+^-free extracellular solution, V_mem_ was ~−25 mV in both CD90^+^ and CD90^−^ cells. Following store depletion, the addition of 1.8 mmol/L extracellular Ca^2+^ induced a strong hyperpolarization in CD90^−^ cells (from −25 ± 3 mV to −67 ± 3 mV, *P* < 0.001), but had no effect on V_mem_ in CD90^+^ cells. The addition of TRAM-34 during SOCE strongly inhibited the SOCE-associated hyperpolarization (Fig. 2d), indicating a key role for *I*_KCa3.1_ in governing V_mem_ in CD90^−^ cells during activation by SOCE.

### *KCNN4*-gene transfer hyperpolarizes V_mem_ and increases intracellular Ca^2+^

**Fig. 3:**
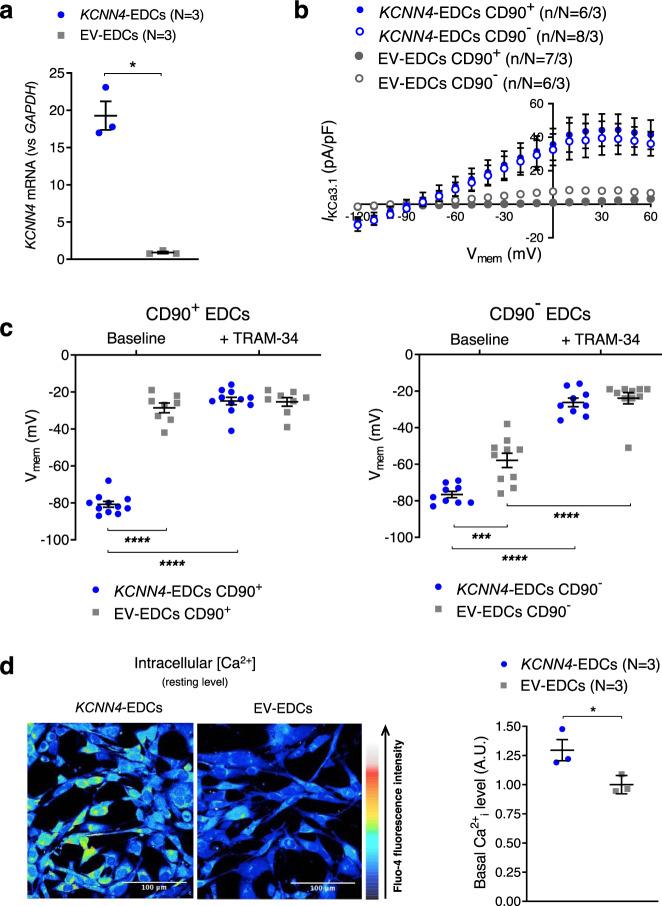
Effect of *KCNN4*-gene transfer on *I*_KCa3.1_ and membrane potential of EDCs. **a** Mean ± SEM *KCNN4*-gene expression in EDCs following lentivirus-mediated *KCNN4*-gene transfer or empty vector (EV; multiplicity of infection = 20). Circles = *KCNN4*-transduced EDCs, squares = EV-EDCs. **b** I–V relationship of *I*_KCa3.1_ currents recorded under various experimental conditions. Blue symbols = *KCNN4*-transduced EDCs, gray symbols = EV-EDCs. Filled symbols = CD90^+^, open symbols = CD90^−^. **c** Resting potential of EDCs under various experimental conditions, before and after exposure to 1 µmol/L TRAM-34. Blue symbols = *KCNN4*-transduced EDCs, gray symbols = EV-EDCs. **d** Representative Fluo-4 images in EV- and *KCNN4*-transduced EDCs overexpression on intracellular [Ca^2+^]. Data in **b**–**d** are shown as individual points, along with mean and SEM. Paired two-sided Student *t*-test; N = biological samples (panel **a**); two-way repeated-measures ANOVA with individual-mean comparisons by Bonferroni-corrected two-sided *t*-tests; n/N = cells/cell lines per group (panel **c**). Blue symbols = *KCNN4*-transduced EDCs, gray symbols = EV-EDCs. Open circles = CD90^−^, closed circles = CD90^+^. EDC explant-derived cell, EV empty vector, min minutes, V_mem_ membrane potential. *P < 0.05, ***P < 0.001, ****P < 0.0001. For precise *P* values, see Supplemental Source Data and Statistics file.

These observations are consistent with the notion that the differential expression of *KCNN4* contributes to the reduced regenerative performance of CD90^+^ cells compared to CD90^−^^11,12^, while providing a potential target to enhance the effectiveness of EDC therapy. We therefore engineered EDCs (mixed CD90^+^/CD90^−^ population) to overexpress KCa3.1 current in EDCs prior to in vivo cell delivery. Lentivirus-mediated *KCNN4*-gene transfer increased *KCNN4*-gene expression about 20-fold compared to the empty vector (EV) (Fig. 3a). *KCNN4* overexpression conferred a much more homogeneous electrophysiological phenotype to EDCs: *KCNN4*-transfer increased *I*_KCa3.1_ density and hyperpolarized V_mem_ to about the same level in both CD90^+^ and CD90^-^ cells (−77 ± 2 mV vs. −81 ± 2 mV; Fig. 3b–c). Because V_mem_ hyperpolarization is known to facilitate Ca^2+^ entry through voltage-independent channels^13^, we investigated whether *KCNN4*-transfer would affect intracellular Ca^2+^. Consistent with our hypothesis, we found that intracellular [Ca^2+^] was significantly higher under resting conditions in *KCNN4*-transferred EDCs compared to EV-control (Fig. 3d). Taken together, these results indicate that KCa3.1 overexpression enhances Ca^2+^ signaling in EDCs.

### Overexpression of *KCNN4* increases proliferation

**Fig. 4:**
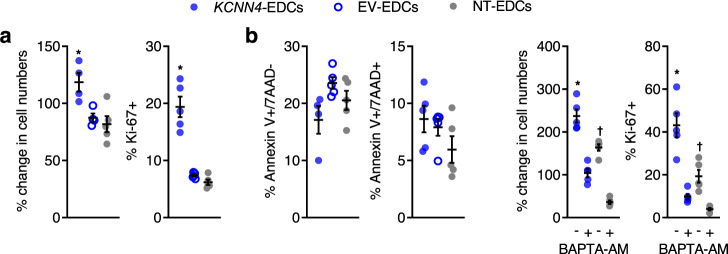
Effect of *KCNN4* overexpression on EDC phenotype after 24 h exposure to ischemia-mimicking media culture conditions. **a** Increasing *I*_KCa3.1_ boosts EDC cell numbers (*n* = 5 biological replicates/group). *P < 0.05 vs. EV- and NT-EDCs. *KCNN4* overexpression increases the proportion of EDCs actively proliferating (*n* = 5 biological replicates/group). *P < 0.05 vs^.^ EV- and NT-EDCs. **b** Increasing *I*_KCa3.1_ had no effect on the ability of EDCs to withstand apoptosis as indicated by expression of apoptotic (Annexin V^+^/7AAD^−^; *n* = 5 biological replicates/group except *KCNN4*-EDCs where *n* = 4 biological replicates) and necrotic (Annexin V^+^/7AAD^+^) markers (*n* = 5 biological replicates). 7AAD 7-aminoactinomycin. **c** Calcium chelation using BAPTA-AM reduces EDC cell numbers (*n* = 5 biological replicates*/*group) and the proportion of EDCs actively proliferating (*n* = 5 biological replicates/group). All data are presented as individual and mean values ± SEM. Significance is indicated in all panels using *P < 0.05 vs. *KCNN4-*EDCs exposed to BAPTA-AM, ^†^*P* < 0.05 vs. NT-EDCs exposed to BAPTA-AM. For precise *P* values, see Supplemental Source Data and Statistics file. All data were analyzed using a one-way ANOVA with individual-mean comparisons by Bonferroni’s multiple two-tailed comparisons test. Blue filled symbols = *KCNN4*-transduced EDCs, blue open symbols = EV-EDCs, gray filled symbols = NT-EDCs. EDC explant-derived cell, EV empty vector.

When EDCs were exposed to in vitro culture conditions designed to mimic the harsh post-infarct environment (1% oxygen/H_2_O_2_/nutrient deprived), both EV- and NT-EDC numbers decreased from baseline, whereas overexpression of *KCNN4* significantly increased proliferation (Fig. 4a and Supplementary Fig. 2a, b) without altering resistance to apoptosis (Fig. 4b and Supplementary Fig. 2c). The importance of intracellular Ca^2+^ in controlling EDC proliferation was underscored by the observation that exposure of *KCNN4*-EDCs to the cell permeant Ca^2+^ chelator BAPTA-AM greatly reduced EDC cell numbers and proliferation (Fig. 4c and Supplementary Fig. 3). Despite ongoing constitutive expression of *KCNN4*, somatic gene transfer did not reduce the cardiogenic potential of EDCs (Supplementary Fig. 4).

### Transplantation of *KCNN4*-engineered EDCs improves cardiac function after myocardial infarction

**Fig. 5:**
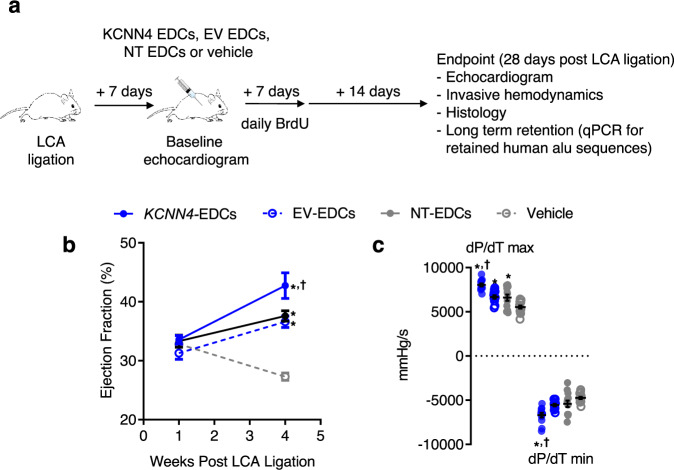
Effects of *KCNN*4 overexpression on myocardial function. **a** Schematic of in vivo experiments comparing the effect of KCNN4-engineered human EDCs (*KCNN4*-EDCs) to empty vector human EDCs (EV-EDCs), nontransduced human EDCs (NT-EDCs) and vehicle using a NOD/SCID IL2Rγ model of ischemic injury. LCA, left coronary ligation. **b** Effects of *KCNN4*-EDCs (*n* = 12 biological replicates), EV-EDCs (*n* = 12 biological replicates), NT-EDCs (*n* = 13 biological replicates), or vehicle (*n* = 14 biological replicates) injection on echocardiographic ejection fraction 4 weeks after LCA ligation. **c** Effects of *KCNN4*-EDCs (*n* = 12 biological replicates), EV-EDCs (*n* = 12 biological replicates), NT-EDCs (*n* = 13 biological replicates) or vehicle (*n* = 14 biological replicates) injection on invasive hemodynamic measures of myocardial function 4 weeks after LCA ligation. All data are presented as individual and mean values ± SEM. Significance is indicated in all panels using *P < 0.0001 vs. vehicle-treated mice, ^†^*P* < 0.05 vs^.^ EV- and NT-EDCs treated mice. For precise *P* values, see Supplemental Source Data and Statistics file. All data was analyzed using a one-way ANOVA with individual-mean comparisons by Bonferroni multiple two-tailed comparisons test. Blue filled symbols = *KCNN4*-transduced EDCs, blue open symbols = EV-EDCs, gray filled symbols = NT-EDCs, gray open symbols = Vehicle. EDC explant-derived cell, EV empty vector.

**Fig. 6:**
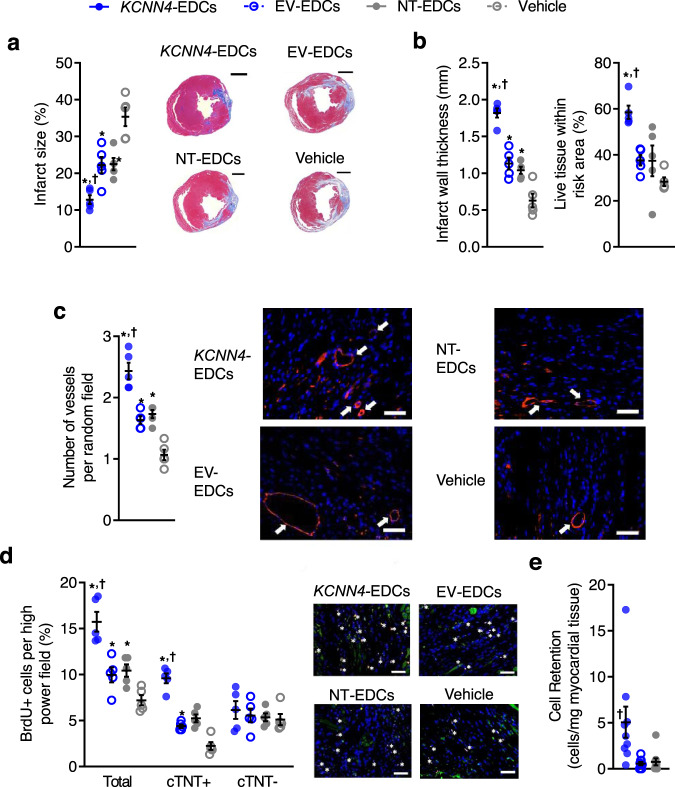
Effects of *KCNN*4 overexpression on infarct properties, neo-angiogenesis, cardiomyogenesis, and long-term engraftment. **a** Scar size analysis 4 weeks after LCA ligation (*n* = 5 biological replicates). Scale bar, 1000 µm. **b** Quantification of infarct wall thickness and viable tissue within the risk area (*n* = 5 biological replicates). **c** Vessel density within the peri-infarct area as indicated (white arrows) using isolectin B4 (red) and 4′,6-diamidino-2-phenylindole (DAPI; blue) immunohistochemistry (*n* = 5 biological replicates). Scale bar, 100 µm. **d** The total number of BrdU positive cells (white stars), proliferating cardiomyocytes (BrdU^+^/cTnT^+^), and noncardiomyocyte cells (BrdU^+^/cTnT^−^) quantified using random field analysis of immunohistochemistry staining with BrdU (red), cTNT (green) and DAPI (blue; *n* = 5 biological replicates). Scale bar, 100 µm. **e** Quantitative PCR analysis for human ALU sequences 21 days after EDC injection (*n* = 9 biological replicates). Significance is indicated in all panels using *P < 0.05 vs. vehicle-treated mice, ^†^*P* < 0.05 vs^.^ EV- and NT-EDCs treated mice. For precise *P* values, see Supplemental Source Data and Statistics file. Data are shown as individual points, along with mean and SEM. All data were analyzed using a one-way ANOVA with individual-mean comparisons by Bonferroni multiple two-tailed comparisons test. Blue filled symbols = *KCNN4*-transduced EDCs, blue open symbols = EV-EDCs, gray filled symbols = NT-EDCs, gray open symbols = Vehicle. EDC explant-derived cell, EV  empty vector, min minutes, V_mem_ membrane potential.

Male NOD/SCID IL2Rγ mice underwent left coronary artery (LCA) ligation, which was followed 1 week later by randomization to echocardiographic guided intramyocardial injection of EV-EDCs, *KCNN4*-EDCs, NT-EDCs, or vehicle (Fig. 5a). Laboratory staff were blinded to treatment allocations and all outcome assessment and analysis was conducted by individuals blinded to group allotment. The study had very little mortality; replacement of subjects was therefore not necessary and not performed (Supplementary Fig. 5). As shown in Fig. 5b and Supplementary Table 2, all animals demonstrated equivalent degrees of pretreatment cardiac dysfunction and chamber dimensions 1 week after LCA ligation. Mice that received EV- or NT-EDCs demonstrated similar improvements in echocardiographic (Fig. 5b and Supplementary Table 2, *P* < 0.05 vs. vehicle) and hemodynamic (Fig. 5b, Supplementary Fig. 6 and Supplementary Table 3) measures of cardiac function 4 weeks after LCA ligation, suggesting that lentiviral transduction per se had no effect on cell-mediated improvement of ischemic injury. Animals that received *KCNN4*-transferred EDCs demonstrated significantly improved cardiac function (Fig. 5b–c, Supplementary Fig. 6 and Supplementary Tables 2 and 3) and showed smaller infarctions (Fig. 6a) 4 weeks after LCA ligation as compared to animals that received vehicle, EV- and NT-EDCs. As shown in Supplementary Fig. 7 and Supplementary Table 4, a separate experimental cohort revealed that the observed differences persisted over the following weeks and did not represent a transient improvement in heart function. Cell treatment had no detectable effect on the ejection fraction of control, noninfarcted mice (Supplementary Fig. 8 and Supplementary Table 5).

Histological analysis revealed that treatment with *KCNN4*-EDCs reduced infarct size (Fig. 6a) and increased both infarct wall thickness and the amount of preserved living tissue within the ischemic area (Fig. 6b). These salutary changes in cardiac structure were paralleled by increases in blood vessel density (Fig. 6c and Supplementary Figs. 9 and 10), healing-promoting M2 macrophages (Supplementary Fig. 11) and proliferative BrdU^+^ cells (Fig. 6d and Supplementary Fig. 12), all of which might have contributed to the reduced expression of apoptotic markers (Supplementary Fig. 13) and of proinflammatory M1 macrophages (Supplementary Fig. 14) seen within the remodeling peri-infarct area. Consistent with the increased in vitro proliferation that we observed, overexpression of *KCNN4* boosted the long-term retention of EDCs by 3 ± 1 (according to human nuclear antigen immunohistochemical profiling; *P* < 0.05, Supplementary Fig. 15) or 7 ± 2 (per retained human alu sequences; *P* < 0.01; Fig. 6e) fold compared to intramyocardial injection of NT-EDCs. By surface telemetry, EDC-transplantation had no effect on ECG-indices of heart rate, cardiac conduction or repolarization (Supplementary Table 6). Programmed electrical stimulation induced ventricular arrhythmias with 2 extrastimuli in a single vehicle-treated animal (Supplementary Fig. 16a) while no EDC-treated animals showed inducible arrhythmias; EDC-transplantation had no detectable effect on cardiac electrophysiology in vivo (Supplementary Fig. 16b). Thus, increasing *I*_KCa3.1_ within EDCs boosts endogenous repair without affecting electrophysiological function or increasing susceptibility to malignant ventricular arrhythmias.

Given that depleting the CD90^+^ fraction enhances the functional benefit of heart-derived cells^11^ and that increasing *I*_KCa3.1_ boosts cell-mediated repair, we hypothesized that the targeted overexpression of *KCNN4* within CD90^+^ EDCs alone might suffice to enhance EDC-mediated repair of injured myocardium. To test this idea, NOD/SCID IL2Rγ mice underwent coronary ligation and were randomized 1 week later to receive intramyocardial injection of *KCNN4* CD90^+^ EDCs, *KCNN4* CD90^-^ EDCs, NT CD90^+^ EDCs, NT CD90^−^ EDCs, recombined KCNN4-EDCs, recombined NT-EDCs or vehicle (Supplementary Figs. 17, 18, and 19; Supplementary Table 7). Interestingly, depletion of CD90^+^ cells alone did not suffice to improve EDC treatment outcomes (NT CD90^+^ cells ≈ NT CD90^−^ cells). Overexpression of *KCNN4* did not significantly improve the regenerative performance of isolated cells, be it CD90^−^ or CD90^+^. Only intramyocardial injection of the recombined KCNN4 cells (i.e., *KCNN4* CD90^−^ and *KCNN4* CD90^+^) improved cell-treatment outcomes, suggesting that important synergies must exist within the mixed cell product that cannot be recapitulated by a single cell type or overcome using genetic engineering. Further work is clearly needed to clarify the underlying mechanisms.

### Overexpression of *KCNN4* increases cytokine and nanoparticle secretion

**Fig. 7:**
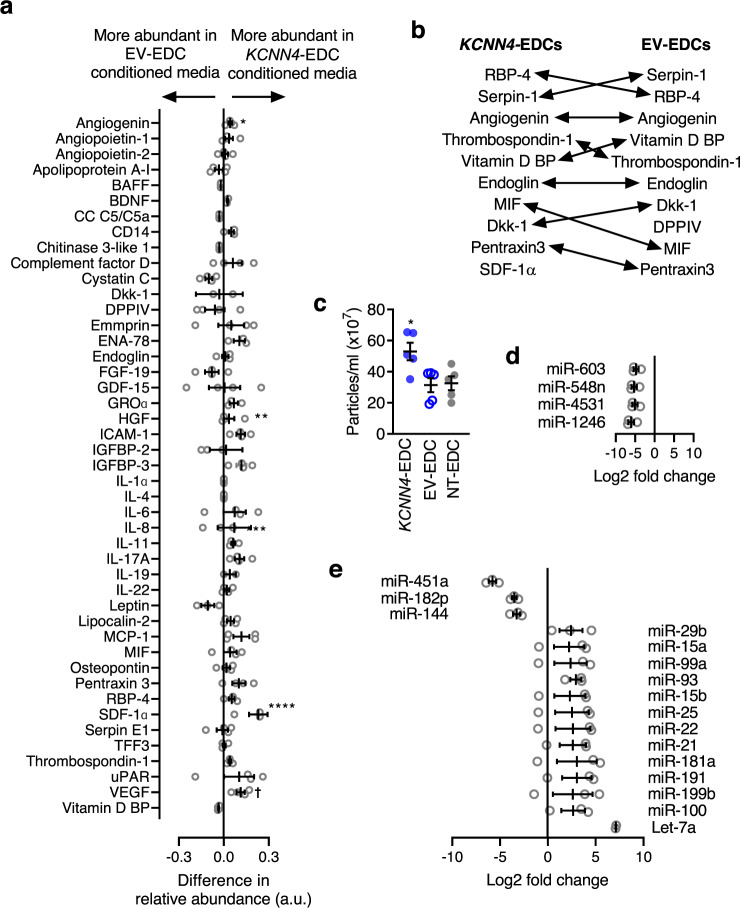
Effects of *KCNN4* overexpression on the paracrine profile of EDCs. **a** Direct comparison of cytokine proteomic expression within 39 cytokines found to be elevated within media conditioned by EV- or *KCNN4*-EDCs. *P < 0.05 vs. EV- or *KCNN4*-EDCs, Fisher’s exact test. BAFF B-cell activating factor, BDNF brain-derived neurotrophic factor, CC C5/C5a complement Component C5a, Dkk-1 Dickkopf-related protein 1, DPPIV Dipeptidyl peptidase-4, ENA-78 epithelial-neutrophil activating peptide, FGF-‘9 fibroblast growth factor 19, GDF-15 growth/differentiation factor 15, GROα growth-regulated oncogene alpha, HGF hepatocyte growth factor, ICAM-1 intercellular adhesion molecule 1, IGBP-2 insulin-like growth factor-binding protein, IGBP-3 insulin-like growth factor-binding protein, IL-6 interleukin-6, IL-8 Interleukin-8, IL-11 interleukin-11, IL-17A interleukin-17A, IL-19 interleukin-19, IL-22 interleukin-22, MCP-1 monocyte chemoattractant protein 1, MIF macrophage migration inhibitory factor, RBP-4 retinol binding protein 4, TFF3 Trefoil factor 3, uPAR urokinase-type plasminogen activator receptor, VEGF vascular endothelial growth factor, Vitamin D BP Vitamin D binding protein. (*n* = 4 biological replicates). Data are shown as mean and SEM. **b** Relative abundance of the top 10 cytokines produced by *KCNN4* and EV-EDCs. **c** Comparison of the size distribution of extracellular vesicles within EDC conditioned media (*n* = 5 biological replicates). Effects of *KCNN4* overexpression relative to the miRNA expression profile of NT-EDCs (**d** *n* = 3 biological replicates) or EV-EDCs (**e** *n* = 3 biological replicates). Data in **c**–**e** are shown as individual points, along with mean and SEM. Significance is indicated in all panels using *P < 0.05 vs^.^ EV- or *KCNN4*-EDCs. For precise *P* values, see Supplemental Source Data and Statistics file. All data were analyzed using a one-way ANOVA with individual-mean comparisons by Bonferroni adjusted two-sided *t*-test. Blue = *KCNN4*-transduced EDCs, gray = EV-EDCs, black = NT-EDCs. EDC explant-derived cell, EV empty vector, min minutes, V_mem_ membrane potential.

The cytokine signature of EDCs was profiled using an unbiased proteomic array capable of detecting 102 cytokines within conditioned media after 48 h of cell culture in nutrient-deprived hypoxic media (1% oxygen) conditions (Fig. 7a). Although *KCNN4* overexpression did not significantly increase the number of cytokines within conditioned media as compared to EV-EDCs (39 vs. 42 cytokines, respectively; Chi-square 0.33, *P* = 0.56), *KCNN4* overexpression increased the production of cytokines already found within EDC conditioned media (6 vs. 1 cytokine levels increased, respectively; Chi-square 3.78, *P* = 0.05). Interestingly, among the cytokines increased via *KCNN4* overexpression several implicated in angiogenesis (vascular endothelial growth hormone)^14,15^, post-infarct healing (angiogenin^16,17^, insulin-like growth factor-binding protein 3^18^, stromal derived factor 1 alpha)^19,20^ and immune modulation (intercellular adhesion molecule 1)^21^ were found (Fig. 7b).

Given recent evidence supporting a critical role for extracellular vesicles in the salutary benefits conferred by heart-derived cell therapy^22^, the effect of *KCNN4* overexpression on extracellular vesicle production was profiled. Media conditioned by *KCNN4*-EDCs demonstrated a 1.6 ± 0.2 fold increase (*P* < 0.05 vs. EV or NT-EDCs; Fig. 7c) in nanoparticles with a size typical for extracellular vesicles (133 ± 5 nm, *P* = ns vs. EV or NT-EDCs). Consistent with previous transcriptome profiling^23^, the most abundant miRNAs found within adherent cultured EDC nanoparticles were associated with cardiomyocyte proliferation (e.g., miR-199a-5p), cardiomyocyte salvage (e.g., miR-125b-5p), protection against oxidative stress (e.g., miR-21-5p), or reducing cardiac fibrosis (e.g., miR-22-3p; Supplementary Table 8). Perhaps surprisingly, somatic gene transfer of *KCNN4* decreased the expression of only 4 miRNAs (*KCNN4*-EDCs vs. NT-EDCs, fold change > 1.5, *P*-adjusted < 0.05, Fig. 7d, Supplementary Table 9). The targets of these miRNAs typically clustered within TGF-β signaling, Hippo signaling, apoptosis, steroid biosynthesis, RNA transport, protein processing, or cytoskeleton regulation (Supplementary Fig. 20a). In comparison to lentiviral transduction alone, *KCNN4* overexpression altered the expression of 16 miRNAs (fold change > 1.5; *P*-adjusted < 0.05, Fig. 7e and Supplementary Table 10) involved in the regulation of stem cell pluripotency, extracellular matrix turnover, cell-cycle regulation, transforming growth factor beta signaling, and Hippo signaling (Supplementary Figs. 20b, c). Thus, akin to effects seen on cytokine production, *KCNN4* activity increases nanoparticle production and alters miRNA expression to improve the healing-promotion signature of the EDC secretome.

## Discussion

In this study, we found that the therapeutic potential of EDCs derived from human hearts is governed by the expression and function of the intermediate-conductance Ca^2+^-activated channel KCa3.1. Although Ca^2+^-activated K^+^ channels were identified in all EDCs, KCa3.1 channels were exclusively expressed in the CD90^-^ subpopulation that is thought to be responsible for most of the functional benefits associated with heart-derived cell therapy^11,12^. Genetic manipulation to enhance KCa3.1-channel expression increased the proliferation and paracrine profile of EDCs in vitro. Compared to nonmodified cells, electrophysiologically engineered EDCs were superior in improving cardiac function in association with a combination of enhanced angiogenesis, myocardial salvage and cardiomyogenesis when transplanted into a clinically relevant mouse model that mirrored post-infarct healing. Our study provides the first evidence in the literature that manipulation of plasma-membrane ion channels can enhance the therapeutic benefits of progenitor cells.

Ion channels provide the basis for generating bioelectric signals that control cell function^24,25^. This important role is underscored by the observation that KCa channels are widely distributed in many adult stem cell types and play a key role during embryonic development^10,26–30^. Until recently, their contribution to the regulation of V_mem_ and Ca^2+^ dynamics has been uncertain—a knowledge gap, which may stem from a myriad of differences in cell culture or technical methods^31^. While BKCa channels have a large conductance, they show strong outward rectification and carry relatively little current in EDCs near the K^+^ reversal potential, which explains why they do not contribute to EDC resting potential (Supplementary Fig. 1).

As shown in mesenchymal stem cells^32–34^, embryonic stem cells^35^ or myoblasts^36,37^, proliferation and differentiation are tightly controlled by changes in V_mem_. In contrast to other cardiac cell types (cardiac fibroblasts^38^ or myocytes)^39^, the membrane potential of EDCs is not determined by Kir-channels. Rather, we found that the EDC resting membrane potential is largely governed by the intermediate-conductance Ca^2+^-activated channel KCa3.1. This finding mirrors our prior observation in expanded bone-marrow-derived mesenchymal stem cells^10^, in which KCa3.1 channels control EDC membrane potential during SOCE to maintain the electrical gradient for Ca^2+^ influx. We now show here that in heart-derived cells, KCa3.1 channels play a pivotal role at the crossroad between Ca^2+^ influx, outward ion fluxes and V_mem_ to provide critical internal signaling, which modulate cell growth, migration, differentiation, and paracrine signaling^40^. When considered within the context of K_Ca_ channel function defining cardiogenesis in both embryonic and induced pluripotent stem cells^41–43^, our results highlight the important physiological role of KCa3.1 regulation of V_mem_ and hint that channel expression may provide an independent phenotypic marker of cell product “potency”.

Somatic gene transfer of *KCNN4* increased proliferation and ultimately improved the long-term engraftment of transplanted cells. Given that *KCNN4* overexpression had negligible effects on resistance to apoptosis, it is unlikely that the acute retention or resistance to toxic metabolites was increased. We hypothesize that the increase in EDC proliferation resulting from *KCNN4*-transfer affords retained human cells the opportunity to multiply and more effectively release cytoprotective molecules. In addition to producing a larger transient pool of transplanted cells within the myocardium that increases paracrine stimulation of endogenous repair mechanisms, long-term cell retention was also greater. However, given the trivial number of transplanted cells that were ultimately retained 3 weeks after injection (597 ± 200 vs. 69 ± 22 for *KCNN4*- vs. NT-EDCs, respectively), it is unlikely that differentiation into working myocardium played any role in the benefits seen after intramyocardial injection.

It is widely agreed that most of the benefits conferred by adult cell therapy occur through paracrine stimulation of angiogenesis, cardiomyogenesis, immunomodulation, and myocardial salvage^5,44^. Consistent with the notion that paracrine engineering enhances cell-treatment outcomes^20,45^, we found that *KCNN4* overexpression improved the paracrine repertoire of EDCs. Since *I*_KCa3.1_-mediated increases in intracellular Ca^2+^ are necessary for the production of inflammatory chemokines and cytokines by various immune cells^46,47^, it is reasonable to believe that a similar mechanism occurs in EDCs. It follows that the marked functional improvement in cardiac function observed after *KCNN4*-EDC transplant is likely mediated, in part, via increased cytokine and nanoparticle stimulation at the site of injection resulting from a combination of increased absolute cell numbers and per-cell paracrine output within the myocardium.

Our findings have potentially significant practical applications, as identifying a heretofore unappreciated mediator of cell transplant outcome has the potential to improve the inconsistent clinical record of cardiac cell therapy^2^. In comparison to expensive, more invasive strategies that repeatedly administer more of the same potentially ineffective cell product, strategies that optimize endogenous ionic-current flows have the potential to regulate more than cell excitability, as evidenced by the marked improvements in cell behavior^48^. Our study is the first to elucidate the important participation of KCa3.1 channels in controlling the therapeutic capacity of progenitor-cell preparations. Given the role of these channels in bone-marrow-derived progenitor cells^10^, our findings may have implications beyond simply cardiac disease. The ability to control cell function by modulating bioelectric properties is a potentially powerful tool to enhance the effectiveness of other progenitor cell types toward therapeutic application.

This is one of the first studies to examine the effect of electrophysiological engineering for cardiac cell therapy. The only prior work in the field that examined the effect of modifying the electrophysiological properties of a cell treatment aimed to reduce arrhythmogenicity (not effectiveness) of therapy with skeletal myoblasts. As these noncardiac cells lack connexin-43, tissue inhomogeneities arise in the form of electrically uncoupled grafts that provide a ready substrate for re-entrant arrhythmias^49^. Their clinical use caused a high incidence of life-threatening ventricular arrhythmias^50–52^. Although therapy to increase expression of connexin-43 increased electrical coupling and decreased arrhythmogenicity^53–55^, equivocal clinical benefits (i.e., no enhancement in left ventricular ejection fraction 6 months after skeletal myoblast injection^56^) combined with ongoing concerns regarding pro-arrhythmia^52^ combined to reduce enthusiasm and stall work on skeletal myoblast-mediated cardiac repair. Unlike the skeletal myoblast experience, our approach focuses on a key regulator, a Ca^2+^-dependent K^+^-current that controls Ca^2+^-entry and cell function, within a cell product undergoing clinical investigation for several diseases (cardiomyopathy secondary to Duchenne Muscular Dystrophy, NCT02485938 & NCT03406780; heart failure with preserved ejection fraction, NCT02941705; pulmonary hypertension, NCT03145298). There has been no signal for adverse electrophysiological effects in the preclinical (50+ independent labs) or clinical (two phase 1, two phase 1/2 and one phase 2) trials, with cardiac EDCs to date. Based on our prior work^10^, we suspect the *KCNN4*-engineering approach may be generalizable to other cell types, but this remains to be shown.

In comparison to other genetically modified cell types used for post-ischemic cardiac repair, several studies have used direct genetic modification to modify stem cell properties before transplantation^57^. One such study demonstrated reduced apoptotic cell clearance and increased long-term retention of cardiac c-Kit^+^ cells by overexpressing a modulator of Akt signaling, Pim-1 kinase^58^. In the past, we have taken a more direct approach by overexpressing cytokines implicated in myocardial repair by direct paracrine engineering^20,45^. Parallel efforts using genetic overexpression within noncardiac cell types have been made to improve cell survival (SDF-1, Bcl-2), differentiation (TGF-β), migration (CXCR4, eNOS), and vasculogenesis (HIF-1, VEGF). *KCNN4* overexpression differs from this previous work as it focuses on a fundamental electrophysiological property, endogenously present within many different types of progenitor cells, that governs replicative function. Boosting electrophysiologic function enhanced proliferation, extracellular vesicle production/potency, and cell-treatment outcomes.

Although *KCNN4*-gene transfer clearly enhanced the therapeutic benefits of cardiac cell therapy, the detailed molecular mechanisms by which boosting KCa3.1-channel-function improved cell function will require further investigation. In this study, we used a viral vector to transfer the genetic material into cells. Lentiviruses are a useful tool for the engineering of adult stem cells because of their transduction efficiency toward slowly proliferating cells, and the absence of detrimental cellular effects^59^. Despite the utility of such vectors to establish proof of concept, integration of genetic material into proto-oncogenic sites is a potential risk associated with the use of retroviruses that limits clinical translation. Other presently available viral vectors will need to be harnessed for clinical translation. This investigation also studied human cells transplanted into immunodeficient mice, making extrapolation to immune competent clearance of allogeneic cells uncertain. Finally, for this proof of principle experiment, we chose to inject cells 1 week after LCA ligation (analogous to ~1 month after a clinical myocardial infarction). Future work will be needed to evaluate the effect of *KCNN4* overexpression on cell-treatment outcomes when therapies are delivered into the extensively scarred and remodeled myocardium.

## Methods

### Cell culture and lentiviral transduction

Human EDCs were cultured from left atrial appendages donated by patients undergoing clinically indicated heart surgery after obtaining informed consent under a protocol approved by the University of Ottawa Heart Institute Research Ethics Board^4^. As shown in Supplementary Table 1, there were no significant differences in the characteristics of patient cell lines used for in vitro or in vivo experiments. Briefly, cardiac biopsies were minced, digested (Collagenase IV, Thermo Fischer Scientific) and plated within MSC Nutristem XF medium (Biological Industries) exposed to physiologic (5%) oxygen^60^. Once a week for 4 weeks, EDCs that spontaneously emerged from the plated biopsy were collected using TrypLE Select (Thermo Fischer Scientific). Cells were transduced with lentivirus to overexpress *KCNN4* (OHS6085-213573573, GE Healthcare Dharmacon), empty backbone (25890, Addgene). Two days later, cells were harvested using TrypLE Select for direct experimentation.

Cardiogenic profiling was performed after exposure to inductive media conditions (Dulbecco’s Modified Eagle Medium Low Glucose, 40% MCDB-201, 0.75% dimethylsulfoxide, 0.1% 10 mmol/l L-ascorbic acid, 0.01% ITS liquid media supplement, 0.01% linoleic acid-albumin, 0.01% Pen-Strep, 0.0002% 0.25 mmol/l dexamethasone, 0.001% 2-mercaptoethanol, 10 ng/ml recombinant mouse fibroblast growth factor 8b, 100 ng/ml fibroblast growth factor 4, 10 ng/ml recombinant human protein rhDKK-1, and 10 ng/ml recombinant human bone morphogenetic protein 2) for 1 week^6,20,61,62^. Total RNA was isolated at day 1 and 7 (Qiagen) for qPCR analysis (Roche) using commercial primers (Integrated DNA Technologies; TNNT2: nm_001001431; ACTA2: nm_001613; VWF: nm_000552). Protein expression was evaluated using flow cytometry for cTNT (ab8295, Abcam), α-SMA, (ab32575, Abcam), VWF (ab8822, Abcam) with appropriate isotype controls (Supplementary Fig. 4). A minimum of 30,000 events was collected with fluorescent compensation performed using single labeled controls (GuavaSoft v2.7, Millipore).

### RNA extraction and gene expression analysis

Messenger RNA were isolated from cultured EDCs using the NucleoSpin RNA Mini kit (Ref 740955.50, Macherey Nagel) and purified mRNA was quantified using a NanoDrop 1000 spectrophotometer. Retrotranscription was performed on 25 ng of RNA with the High-Capacity cDNA Reverse Transcription Kit (Ref 4368814, Thermofisher) using standard protocol (Annealing 10 min 25 °C, Elongation 2 h 37 °C, 10 sec 85 °C). RT-qPCR was done using SyBR Green primers for GAPDH and Kcnn4 (Supplementary Table 11). SyBr Select Master Mix (Ref 4472903, Thermofisher) was added to primers and 1 ng cDNA per well. RT-qPCR was run on a StepOnePlus™ Real-Time PCR System (4376600, Thermofisher) with an initial denaturation 95 °C step for 10 min, followed by 40 cycles (Denaturation 95 °C 15 sec—Annealing and Elongation 60 °C for 1 min). The Kcnn4 Ct values were normalized to GAPDH and data were analyzed using the 2^-ΔCt^ method.

### Ion-current and V_mem_ recording

All in vitro recordings were obtained at 37 °C. Whole-cell perforated-patch and tight-seal techniques were used to record membrane potential (V_mem_, current-clamp mode) and ion-currents (voltage-clamp mode). Borosilicate glass electrodes (tip resistances 2–3 MΩ when filled with pipette solution) were connected to a patch-clamp amplifier (Axopatch 200B; Axon Instruments). *I*_KCa3.1_ was recorded as 1 µmol/L TRAM-34–sensitive current, and I_BKCa_ as 1 µmol/L paxilline-sensitive current. Tyrode solution contained (mmol/L) NaCl 136, CaCl_2_ 1.8, KCl 5.4, MgCl_2_ 1, NaH_2_PO_4_ 0.33, dextrose 10, and HEPES 5, titrated to pH 7.4 with NaOH. For nominally Ca^2+^-free Tyrode solution experiments, CaCl_2_ was omitted and 1 mM EGTA was added. The pipette solution used to define global ion-current profiles contained (mmol/L) GTP 0.1, potassium-aspartate 110, KCl 20, MgCl_2_ 1, MgATP 5, HEPES 10, sodium-phosphocreatine 5, and EGTA 5 (pH 7.4, KOH). For *I*_KCa3.1_ recording, the pipette solution contained (mmol/L) KCl 130, NaCl 5, MgCl_2_ 1, K_2_ATP 5, HEPES 10, and EGTA 5 (pH 7.2, KOH). The amount of CaCl_2_ required to achieve 300 nmol/L free-[Ca^2+^]_i_ was determined with WebMaxC standard software (http://www.stanford.edu/∼*cpatton/webmaxc/webmaxcS.htm*). Junction potentials between bath and pipette solution averaged 10 mV and were corrected before recording, for both V_mem_ and ion-current measurements. Currents are expressed as densities (pA/pF) to control for cell size/capacitance differences. To study the effect of SOCE on V_mem_, Ca^2+^_i_ was depleted passively by bathing cells in Ca^2+^-free Tyrode solution for at least 30 min, and then cell contents were dialyzed by tight-seal attachment with pipette solutions containing (mmol/L) KCl 140, NaCl 5, MgCl_2_ 1.2, and HEPES 10 (pH 7.2, KOH). After whole-cell configuration and cell-dialysis were established, extracellular [Ca^2+^] was restored and V_mem_ changes were recorded. Before starting patch-clamp experiments, a fluorescently-tagged antibody (FAB2067G, R&D Systems) was used to labeled the CD90^+^ subpopulation.

### Ca^2+^-imaging

EDCs genetically engineered to overexpress *KCNN4* (*KCNN4*-EDCs) and empty backbone-transduced EDCs (EV-EDCs) were loaded with Fluo-4-acetoxymethyl ester (10 μmol/L; Invitrogen) in complete growth medium in the presence of Pluronic F-127 (20% solution in dimethylsulfoxide, 2.5 μg/mL) for 30 min at 37 °C in a humidified incubator under standard cell culture conditions. Chamber slides were positioned on the stage of a confocal microscope; cells were incubated with Tyrode solution and maintained for 15 min at room temperature before experimental protocols to allow deesterification of Fluo-4-acetoxymethyl ester. Fluo-4 was excited at 488 nm; emitted fluorescence was collected at 495 nm. High power field images were acquired with a confocal microscope (Olympus IX81). Basal intracellular Ca^2+^ level was assessed in both groups. Images were analyzed with ImageJ software. Data are presented as mean fluorescence intensity relative to EV-EDCs.

### Colorimetric, flow cytometric, immunohistochemical, proteomic, and nanoparticle evaluation

EDC proliferation was measured in conditions designed to mimic the ischemic environment of the heart (1% oxygen HERAcell 150i, Thermo Scientific; basal media without growth factors)^63,64^ and in the presence of BAPTA-AM (B1205, Fisher). Relative cell counts were evaluated using a colorimetric WST-8 assay (Cell Counting Kit-8, Dojindo) with confirmatory manual cell counts and random field analysis for Ki67/DAPI expression (12 visual field per-cell line; ab156956, Abcam). After 18 h of culture in 1%-oxygen basal media without growth factors conditions, EDCs and the culture medium were collected for analysis. Flow cytometry (Guava easyCyte, EMD Milipore) was used to evaluate cell viability with phycoerythrin-Annexin 5 (PE-Annexin V) and 7-Aminoactinomycin D (7AAD) (559763, BD Biosciences). The relative abundance of select cytokines within conditioned media was evaluated with Proteome Profiler Human XL Cytokine Array Kit (R&D Systems). To isolate extracellular vesicles, conditioned media were collected after culturing cells for 48 h in 1% exosome-free serum (System Biosciences) and 1% oxygen culture conditions. Briefly, conditioned media were first centrifuged at 8000 × *g* for 10 min at 4 °C (5810-R centrifuge; Eppendorf) to remove dead cells, cocoons and large debris, followed by centrifugation at 10,000 × *g* for 30 min at 4 °C (Optima XE-90 ultracentrifuge; Beckham Coulter) to remove large vesicles and debris. The supernatant was than centrifuged at 100,000 × *g* for 2 h at 4 °C to pellet extracellular vesicle. Nanoparticle Tracking Analysis (Nanosight V2.3) was used to quantify extracellular vesicle content within conditioned media followed by multiplex fluorescent oligonucleotide-based miRNA detection (Human v3, Nanostring) to miRNA content within extracellular vesicles. Briefly, miRNeasy Micro Kit (Qiagen) was used to extract total RNA with RNA quality/quantity measured Agilent 2100 Bioanalyzer (Agilent). Twenty-five nanograms of RNA were used for each reaction (Counter Human V3 miRNA Expression Assay, Nanostring). Image-quality control metrics were evaluated with nSolver (Nanostring); background subtraction was performed with the mean of negative controls plus two standard deviations. Normalized counts were obtained with trimmed-mean of M values (TMM)64 and differentially expressed miRNA identified with the generalized linear model (GLM) likelihood-ratio-test65 using EdgeR in the online DEBrowser tool (https://debrowser.umassmed.edu/). Heat maps were created in DEBrowser tool using the “complete” clustering method.

### In vivo protocol

This protocol was reviewed and approved by the University of Ottawa Animal Care Committee. The detailed protocol was registered a priori within the Open Science Framework (https://osf.io/nx2ck/). Male NOD/SCID IL2Rγ mice (8–9 weeks old; Charles River) were pretreated with buprenorphine and anaesthetized with isoflurane under normothermic temperature control, for surgical LCA ligation. Seven days after LCA ligation, animals were randomized to echocardiographic guided intramyocardial injection of 100,000 unmodified EDCs (NT-EDCs), 100,000 *KCNN4*-EDCs, 100,000 EV-EDCs or saline (i.e., vehicle-treated group) divided into two 14 μl injections at the apex and ischemic border zone^6–8,20,23,45,65^. During the surgery and functional evaluation, mice were intubated, anesthetized with 2–3% isoflurane and maintained under physiologic temperature control. All animals were injected with buprenorphine (0.05 mg/kg subcutaneous) 1 h prior to surgery and twice daily thereafter for 3 days. All mice were injected with bromodeoxyuridine (BrdU, 100 mg/kg IP daily) for 1 week after cell/vehicle injection.

Laboratory staff were blinded to treatment allocations and all outcome assessment and analysis was conducted by individuals blinded to group allotment. Group allocations were kept in a separate password protected list for unblinding after analysis of functional study outcome was completed. All mice underwent echocardiographic imaging to confirm the effects of LCA ligation (1-week post LCA ligation) and cell therapy (21 days post-cell/vehicle injection). Twenty-eight days after cell/vehicle injection, mice underwent invasive hemodynamics or invasive electrophysiological study prior to sacrifice. Mice randomized to invasive hemodynamics underwent insertion of a 1.2 F Millar catheter into the left ventricle via the right carotid artery. Transient inferior vena cava (IVC) occlusion to reduce preload was used to change the loading conditions and generate pressure-volume loops. Mice randomized to invasive electrophysiological testing underwent a thoracotomy to expose the apex of the heart prior to programmed electrical stimulation (MyoPacer EP, Ion Optix) via a platinum electrode placed on the apex of the left ventricle^66^. A standard programmed electrical stimulation protocol was performed, consisting of 10 stimuli delivered at 100 ms intervals (S1, twice threshold, 2 ms) followed by a single extrastimulus (S2) starting at a coupling interval of 80 ms which then decremented by 2 ms until failure to capture defined the effective refractory period (ERP). If ventricular tachycardia or fibrillation were not induced, a second extrastimulus (S3) was introduced 80 ms after the shortest S2 that captured the ventricle. The S3 was then progressively decremented by 2 ms intervals until the ERP was reached. Finally, a third extrastimulus (S4) was introduced 80 ms after the last S3 that captured the ventricle and was then decremented by 2 ms intervals until the ERP was reached. If the mouse failed to develop ventricular arrhythmias with extrastimuli, the animal was deemed noninducible. Infarct size was quantified using histological sections stained with Masson-Trichrome (ThermoFisher) in which sections at equivalent distances from the LCA surgical stitch were directly compared. Infarct wall thickness was defined as the average of 5 left ventricular wall thickness measurements distributed equally within the infarcted left ventricular scar, while the ischemic risk region was defined as the area between the two edges of the infarct scar^67^. Adjacent sections were used for immunohistochemical detection of capillary density (isolectin B4, B-1205, Vector Laboratories), endogenous proliferation (BrdU, ab6326, Abcam), CD68+ cells (ab955, Abcam), CD163+ cells (ab182422, Abcam), or cleaved caspase 3 cells (9664 S, Cell Signaling Tech) in conjunction with DAPI (Sigma–Aldrich) or cardiac troponin-T (cTnT, ab125266; Abcam) labelling. Human-cell engraftment was quantified using quantitative polymerase chain reaction for retained human alu sequences (Supplementary Table 11) and immunohistochemical analysis for human nuclear antigen (mab1281, Sigma–Aldrich) expression^6–8,20,23,45^.

To evaluate the effect of EDCs on the ejection fraction of control noninfarcted mice, a separate cohort of control noninfarcted NOD/SCID IL2Rγ mice were randomized to intramyocardial injection of 100,000 NT-EDCs, 100,000 *KCNN4*-EDCs or saline solution. The ability of treatment effects to persist beyond the 21-day post-injection endpoint was investigated in a separate cohort of NOD/SCID IL2Rγ mice randomized 1 week after LCA ligation to echocardiographic guided intramyocardial injection of 100,000 unmodified EDCs (NT-EDCs), 100,000 *KCNN4*-EDCs, 100,000 EV-EDCs or saline (i.e., vehicle-treated group). All animals underwent echocardiographic evaluation to confirm the effects of LCA ligation (1-week post LCA ligation) while the effects of cell therapy were evaluated 21 (control noninfarcted mice) or 56 (infarcted long-term study mice) days after cell/vehicle injection.

The effect of targeted *KCNN4* overexpression within CD90^−^ cells alone was evaluated in a separate series of NOD/SCID IL2Rγ mice that underwent coronary artery ligation and were then randomized 1 week later to echocardiographically guided intramyocardial injection of 100,000 *KCNN4* CD90+ EDCs, 100,000 *KCNN4* CD90^−^ EDCs, 100,000 NT CD90+ EDCs, 100,000 NT CD90^−^ EDCs, 100,000 KCNN4-EDCs, or 100,000 NT-EDCs. Magnetically activated cell sorting was performed using anti‐CD90 microbeads (555596; BD Biosciences) and CD90+ cells were cultured in parallel from the CD90-depleted fraction. Cells transduced with *KCNN4* were recombined after 48 h. Importantly, both *KCNN*4 and NT-EDCs underwent magnetic separation and parallel cell culture prior to recombination to control for effects attributable to magnetic separation. All animals underwent echocardiographic imaging 7 and 28 days after LCA prior to sacrifice for histological evaluation of infarct size (Mason-Trichrome).

### Statistical analysis

Clampfit 10.4 (Axon Instruments) and GraphPad Prism 6.0 were used for data analysis. Experiments and analyses were performed blinded to treatment allocation. Animals were allocated randomly to experimental groups as follows: a series of sealed envelopes were prepared that contained allocation to each treatment. Envelopes were evenly distributed throughout the surgery days. Four to eight surgeries were performed each day. Lab staff prepared 1–2 treatments of each type (e.g., *KCNN4*-EDCs, EV-EDCs, NT-EDCs, or vehicle) per lab day. The animal surgeon opened the envelope and administered the treatment. All data are presented as mean ± SEM. All statistical comparisons were with two-sided tests. Multiple group comparisons were obtained with one-way ANOVA for nonrepeated analyses in experiments involving more than two groups and two-way repeated-measures ANOVA for all multi-group analyses involving repeated measures. In all cases, normality was confirmed prior to further post-hoc testing. If ANOVA showed significant differences, Bonferroni’s multiple comparisons test was used to compare individual means. Differences in categorical measures were compared using Fischer’s exact test. *P* < 0.05 was considered statistically significant. All raw data supporting the findings from this study are available from the corresponding authors upon reasonable request.

### Reporting summary

Further information on research design is available in the Nature Research Reporting Summary linked to this article.

## Supplementary information

Supplementary Information

Reporting Summary

## Data Availability

The data supporting the findings from this study are available within the article and its supplementary information. Any remaining raw data will be available from the corresponding authors upon reasonable request. Source data are provided with this paper.

## Source data

Source data
