# Supplementary material for: Electrophysiological engineering of heart-derived cells with calcium-dependent potassium channels improves cell therapy efficacy for cardioprotection
Source: Nat Commun. 2021 Aug 16;12:4963. doi: 10.1038/s41467-021-25180-8 (PMC8368210; doi:10.1038/s41467-021-25180-8)
Supplement: Supplementary file 1 — Supplementary Information [file 41467_2021_25180_MOESM1_ESM.pdf]

Supplementary Materials for

Electrophysiological engineering of heart-derived cells for regenerative-therapy: Calcium-dependent potassium-channels govern cell-therapy efficacy for cardioprotection

Patrick Vigneault, Sandrine Parent, Pushpinder Kanda, Connor Michie, Darryl R. Davis*, Stanley Nattel*

*Corresponding author. Email: ddavis@ottawaheart.ca (D.R.D.); stanley.nattel@icm-mhi.org (S.N.)

This PDF file includes:

SUPPLEMENTARY FIGURES.. p.3

Supplementary Fig. 1. Functional BKCa current in EDCs. a *I-V* relationship of I_{BKCa} (paxilline–sensitive current) in $CD90^+$ and $CD90^-$ EDCs.
Supplementary Fig. 2. Effect of *KCNN4* engineering on EDC proliferation.
Supplementary Fig. 3. Effect of calcium chelation on KCNN4 mediated increases in proliferation.
Supplementary Fig. 4. Effect of *KCNN4* engineering on EDC expression of cell-type selective markers.
Supplementary Fig. 5. Group allocation, outcomes and endpoints for the in vivo 4 week study.
Supplementary Fig. 6. Hemodynamic effects of transplanting *KCNN4* engineered EDCs.
Supplementary Fig. 8. Effect of cell injection on normal heart function. No significant difference was detected.
Supplementary Fig. 9. Representative images demonstrating vessel density within the peri-infarct region 4 weeks after LCA ligation.
Supplementary Fig. 10. Effect of KCNN4 over-expression on peri-infarct vascularization.
Supplementary Fig. 11. Effect of KCNN4 over-expression on pro-healing M2 macrophages.
Supplementary Fig. 12. Representative images demonstrating $BrdU^+/DAPI^+$ cells within the peri-infarct region 4 weeks after LCA ligation.
Supplementary Fig. 13. Effect of KCNN4 over-expression on markers of apoptosis.
Supplementary Fig. 14. Effect of KCNN4 over-expression on cardiac macrophage numbers
Supplementary Fig. 15. Effect of KCNN4 over-expression on EDC engraftment.
Supplementary Fig. 16. Electrophysiological effects of EDC or vehicle treatment on mice.
Supplementary Fig. 17. In vivo study exploring the impact of KCNN4 over-expression on EDC mediated repair.
Supplementary Fig. 18. Group allocation, outcomes and endpoints for in vivo recombination cell product study.
Supplementary Fig. 19. In vivo study exploring the impact of KCNN4 over-expression on EDC mediated repair.
Supplementary Fig. 20. KEGG pathway analysis for molecular pathways predicted to be affected by miRNAs altered by *KCNN4* overexpression

SUPPLEMENTARY TABLES.. p.25

Supplementary Table 1. Clinical characteristics of atrial appendage donors used to obtain EDCs.
Supplementary Table 2. Echocardiographic measures of left ventricular function.
Supplementary Table 3. Invasive hemodynamic measures of left ventricular function.
Supplementary Table 4. Echocardiographic measures of left ventricular function examining the long-term durability of cell transplantation on heart function.
Supplementary Table 5. Echocardiographic measures of left ventricular function examining the effect of cell injection on normal heart function.
Supplementary Table 6. Effect of *KCNN4* overexpression on electrophysiological parameters.
Supplementary Table 7. Echocardiographic measures of left ventricular function examining the impact of KCNN4 over-expression on EDC mediated repair.
Supplementary Table 8. Top 10 miRNAs within adherent explant-derived cell extracellular vesicles involved with cardiomyocyte proliferation, salvage, and modulating cardiac fibrosis.
Supplementary Table 9. Effect of *KCNN4* overexpression on the miRNA profile within extracellular vesicles compared to non-transduced explant-derived cells.
Supplementary Table 10. Effect of *KCNN4* overexpression on the miRNA profile within extracellular vesicles compared to empty vector explant-derived cells.

SUPPLEMENTARY REFERENCES... p.38

SUPPLEMENTARY FIGURES

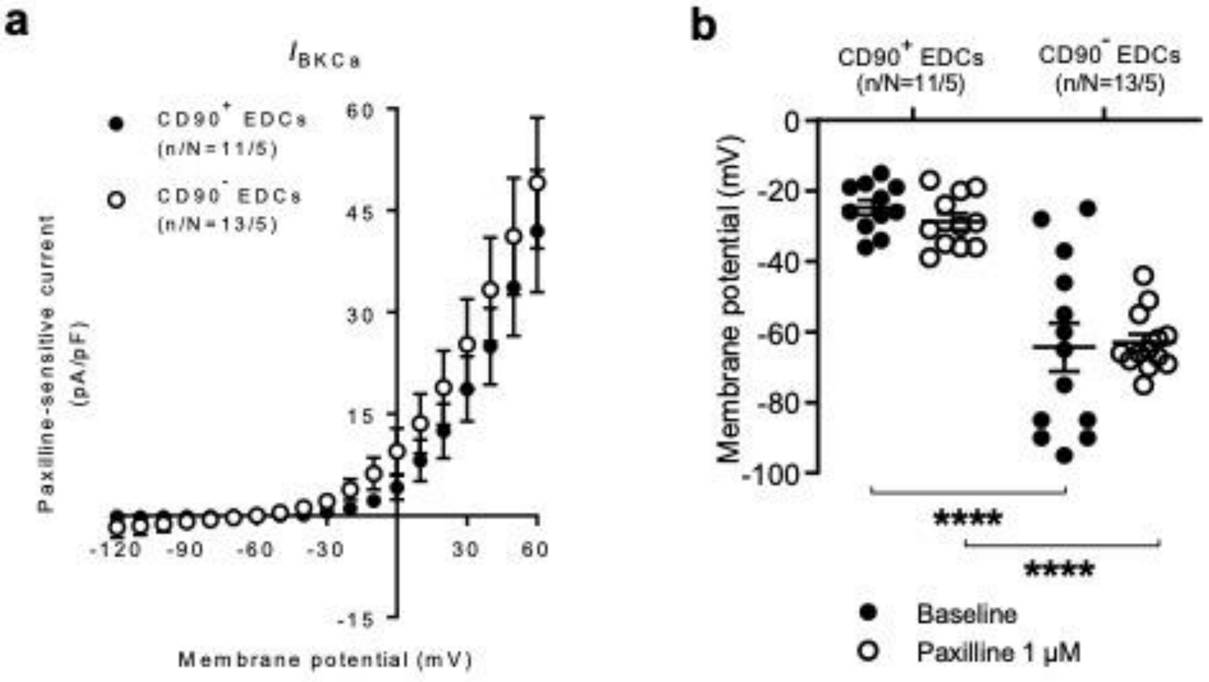

Supplementary Fig. 1. Functional BKCa current in EDCs. **a.** *I-V* relationship of I_{BKCa} (paxilline–sensitive current) in CD90^{+} and CD90^{-} EDCs. **b.** Resting potential of EDCs before and after exposure to 1-µmol/L paxilline. Data are shown as individual data points from biologically-independent experiments, as well as mean and SEM. Two-way repeated-measures ANOVA with individual-mean comparisons by Bonferroni-corrected t-tests; n/N = cells/cell lines per group. For precise P values, see Supplemental Excel file**.** ****$P < 0.0001$.

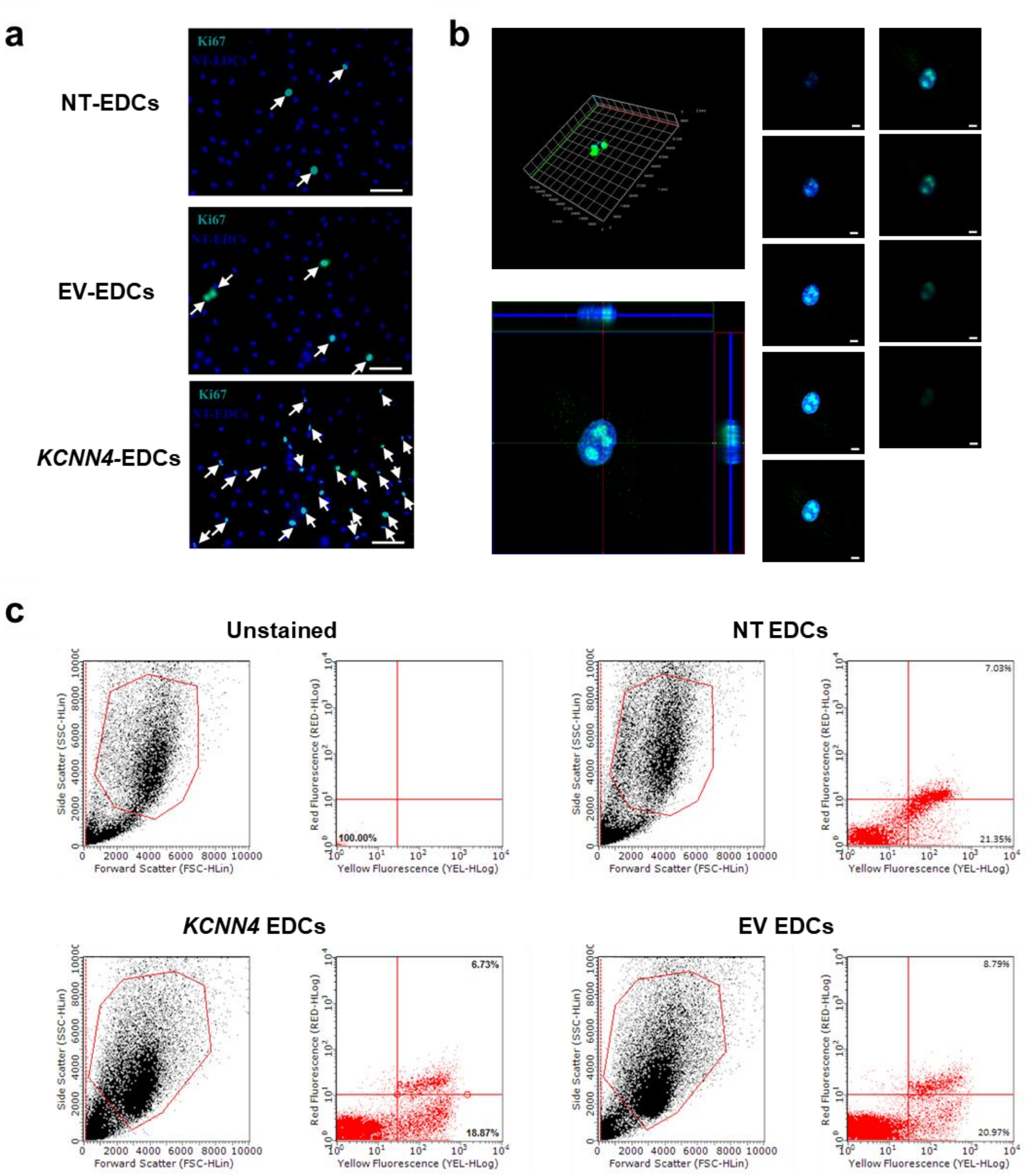

Supplementary Fig. 2. Effect of *KCNN4* engineering on EDC proliferation and apoptosis. **a.** Representative images showing the expression of the proliferative marker Ki67. Arrows indicate Ki67⁺ cells. Scale bar, 100 µm. The experiment was independently repeated in separate biological experiments 5 times for each cell type with similar results. **b.** Representative three-dimensional reconstruction of z-stacked confocal images of EDCs showing DAPI (blue) and Ki-67 (green) (upper panel). Ortho-representation of z-stack confocal microscope images to confirm co-localization of Ki-67 stain (green) within nucleus (DAPI; blue) (lower panel). Nine separate stacked images to show Ki-67 marker (green) localized inside the nucleus of the cell (blue) (right panels). Scale bar, 5 um. DAPI, 4′,6-diamidino-2-phenylindole. c. Representative images showing the flow cytometry gating strategies used to quantify cells stained with Annexin V-PE (yellow fluorescence, x axis) and cells stained with 7-AAD (red fluorescence, y axis).

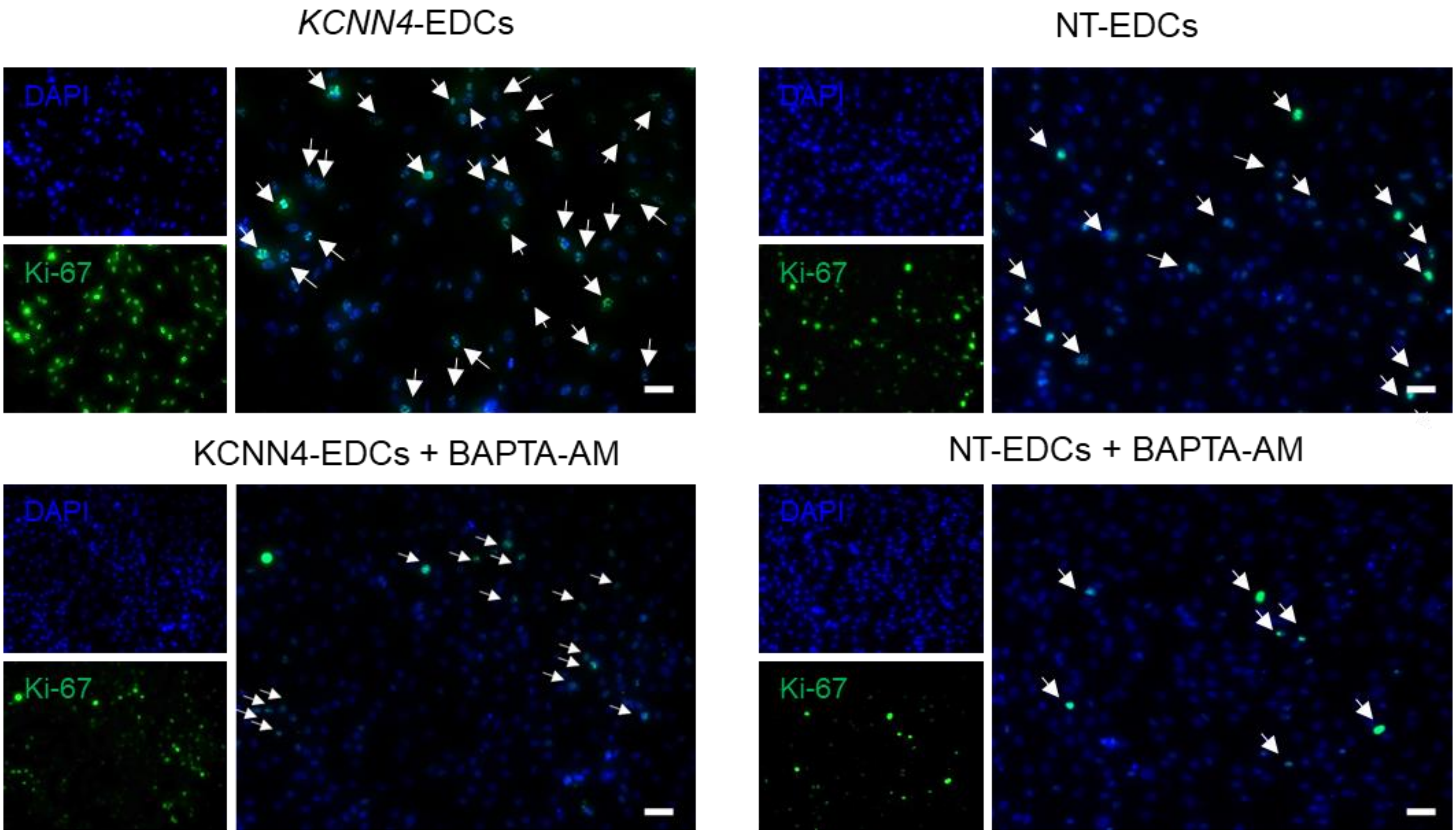

Supplementary Fig. 3. Effect of calcium chelation on KCNN4 mediated increases in proliferation. Representative images demonstrating Ki-67 (green) /DAPI (blue) positive cells. Scale bar, 100 um. The experiment was independently repeated in separate biological replicates 5 times for each cell type with similar results. Arrows point to Ki-67+ cells.

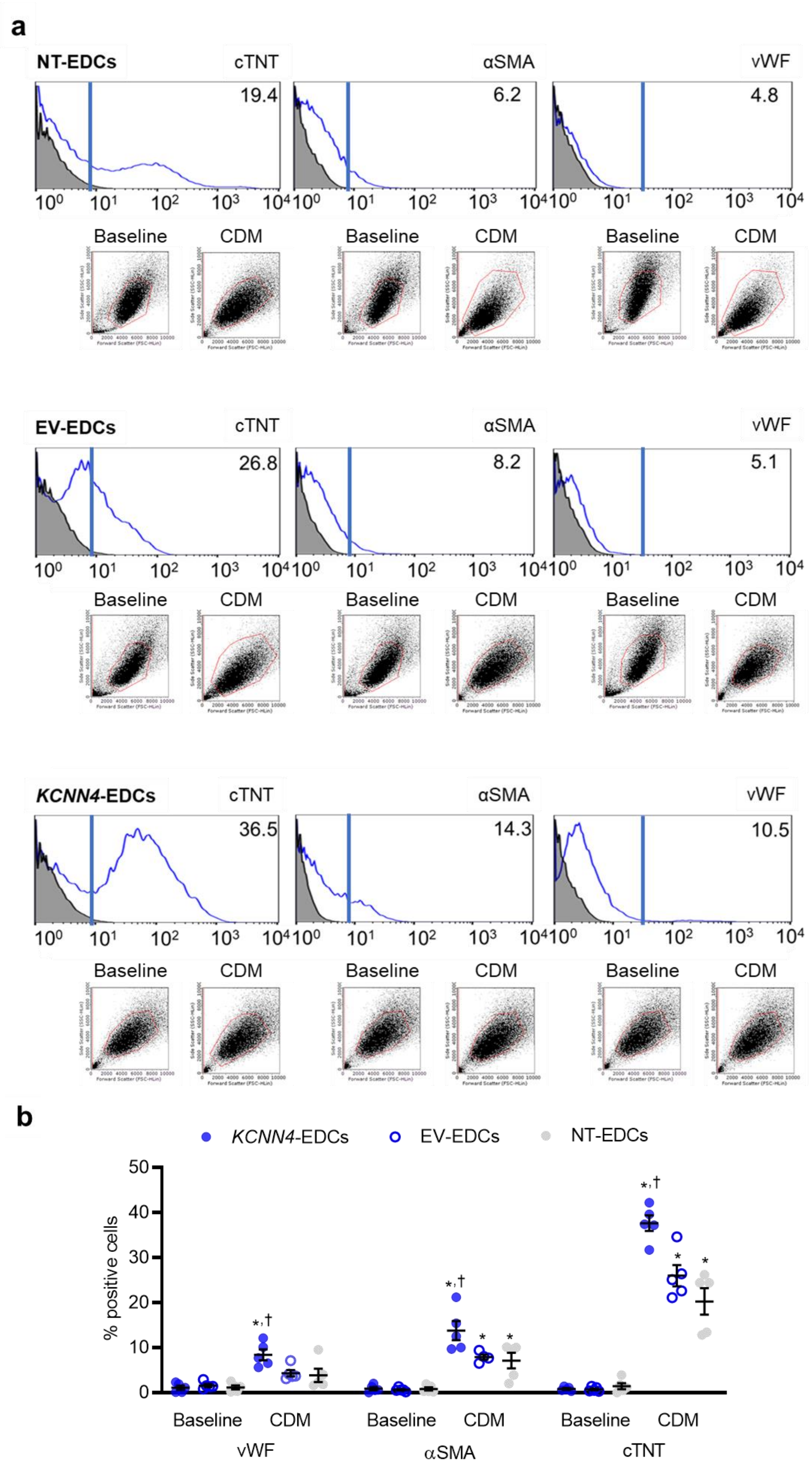
a
NT-EDCs
cTNT
19.4
αSMA
6.2
vWF
4.8
EV-EDCs
cTNT
26.8
αSMA
8.2
vWF
5.1
KCNN4-EDCs
cTNT
36.5
αSMA
14.3
vWF
10.5
10^0 10^1 10^2 10^3 10^4
Baseline
CDM
b
KCNN4-EDCs
EV-EDCs
NT-EDCs
% positive cells
50
40
30
20
10
0
*,†
*
Baseline
CDM
vWF
αSMA
cTNT

Supplementary Fig. 4. Effect of *KCNN4* engineering on EDC expression of cell-type selective markers. **a.** Representative images of flow cytometry plots for cardiac troponin T (cTNT, selectively expressed by cardiomyocytes), alpha smooth muscle actin (αSMA, typically expressed in vascular smooth-muscle cells) and von Willebrand factor (vWF, expressed in endothelial cells), before and after 1 week of culture of non-transduced (NT)- explant-derived cells (EDCs), empty-virus (EV)-EDCs and *KCNN4*-gene transferred EDCs (*KCNN4*-EDCs) in cardiogenic differentiation media (CDM). **b.** Mean+SEM data demonstrating the effect of *KCNN4* overexpression on the expression pattern of EDCs (n=5 biological repeats for all groups except EV-EDCs treated with cardiogenic media and stained for alpha smooth muscle actin where n = 4). *$P < 0.05$ vs. baseline. †$P < 0.05$ vs. EV-EDCs and NT-EDCs after 1 week of culture within CDM. For precise P values, see Supplemental Excel file. All data was analyzed using a one-way ANOVA with individual-mean comparisons by Bonferroni multiple two-tailed comparisons test.

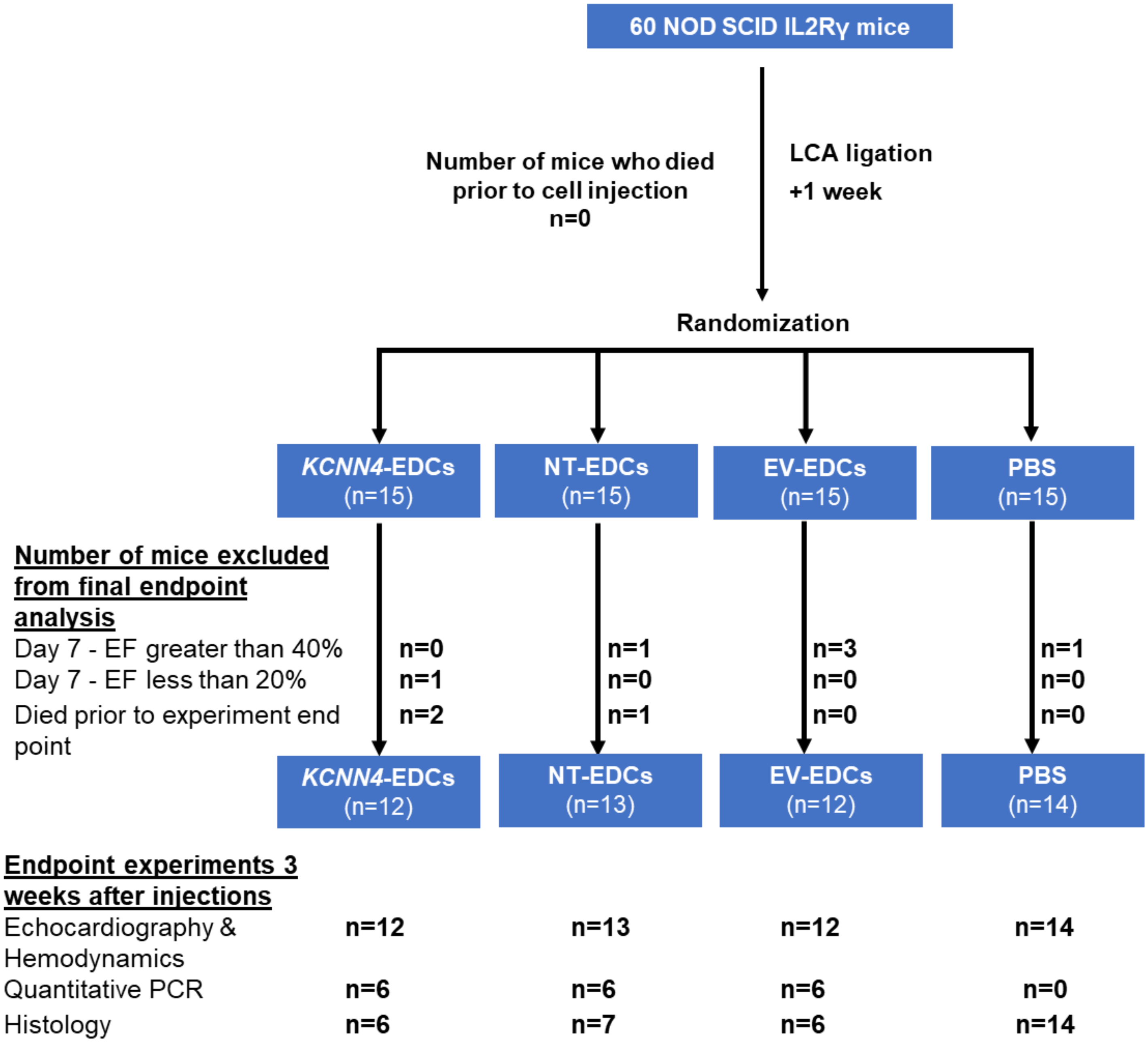

Supplementary Fig. 5. Group allocation, outcomes and endpoints for the in vivo 4 week study.

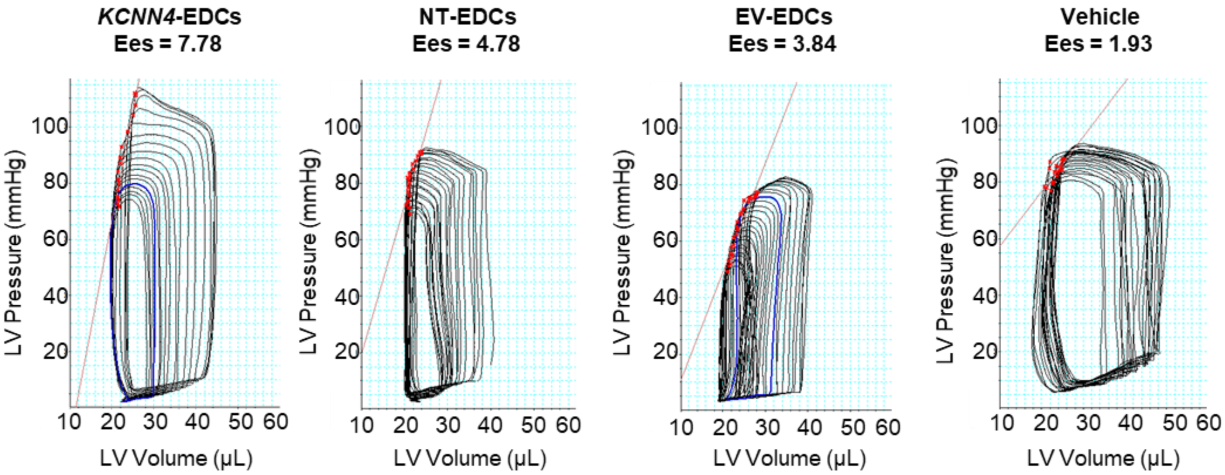

Supplementary Fig. 6. Hemodynamic effects of transplanting *KCNN4* engineered EDCs. Representative images of pressure-volume loops generated during IVC compression from mice 3 weeks after receiving *KCNN4*-EDCs, EV-EDCs, NT-EDCs or vehicle. Ees = end-systolic elastance.

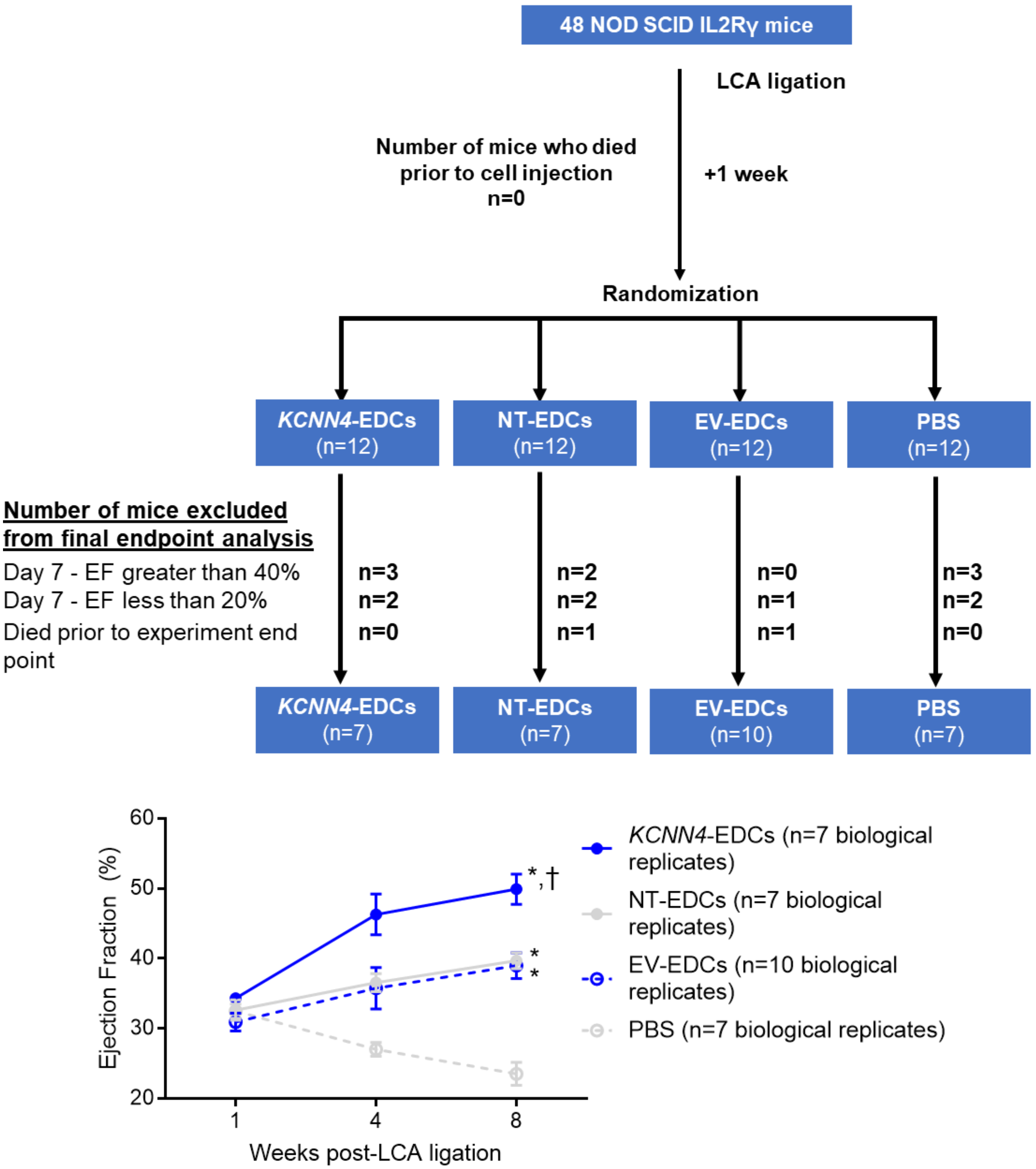

Supplementary Fig. 7. Long-term durability of cell transplantation on heart function. *$P < 0.05$ vs. vehicle treated mice, †$P < 0.05$ vs. EV- or NT-EDCs treated mice. Data are shown as mean and SEM. For precise P values, see Supplemental Excel file. All data was analyzed using a one-way ANOVA with individual-mean comparisons by Bonferroni multiple comparisons test.

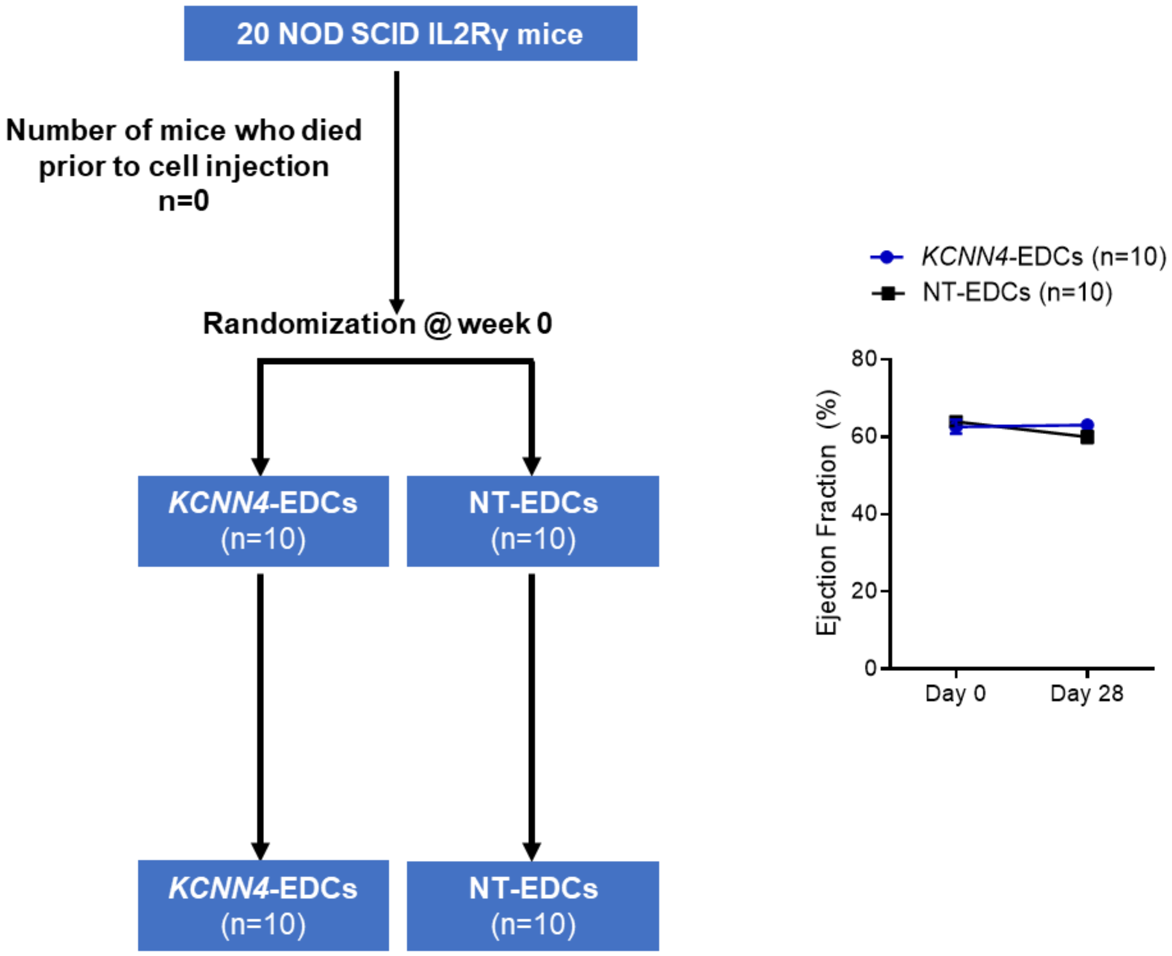

Supplementary Fig. 8. Effect of cell injection on normal heart function. No significant difference was detected.

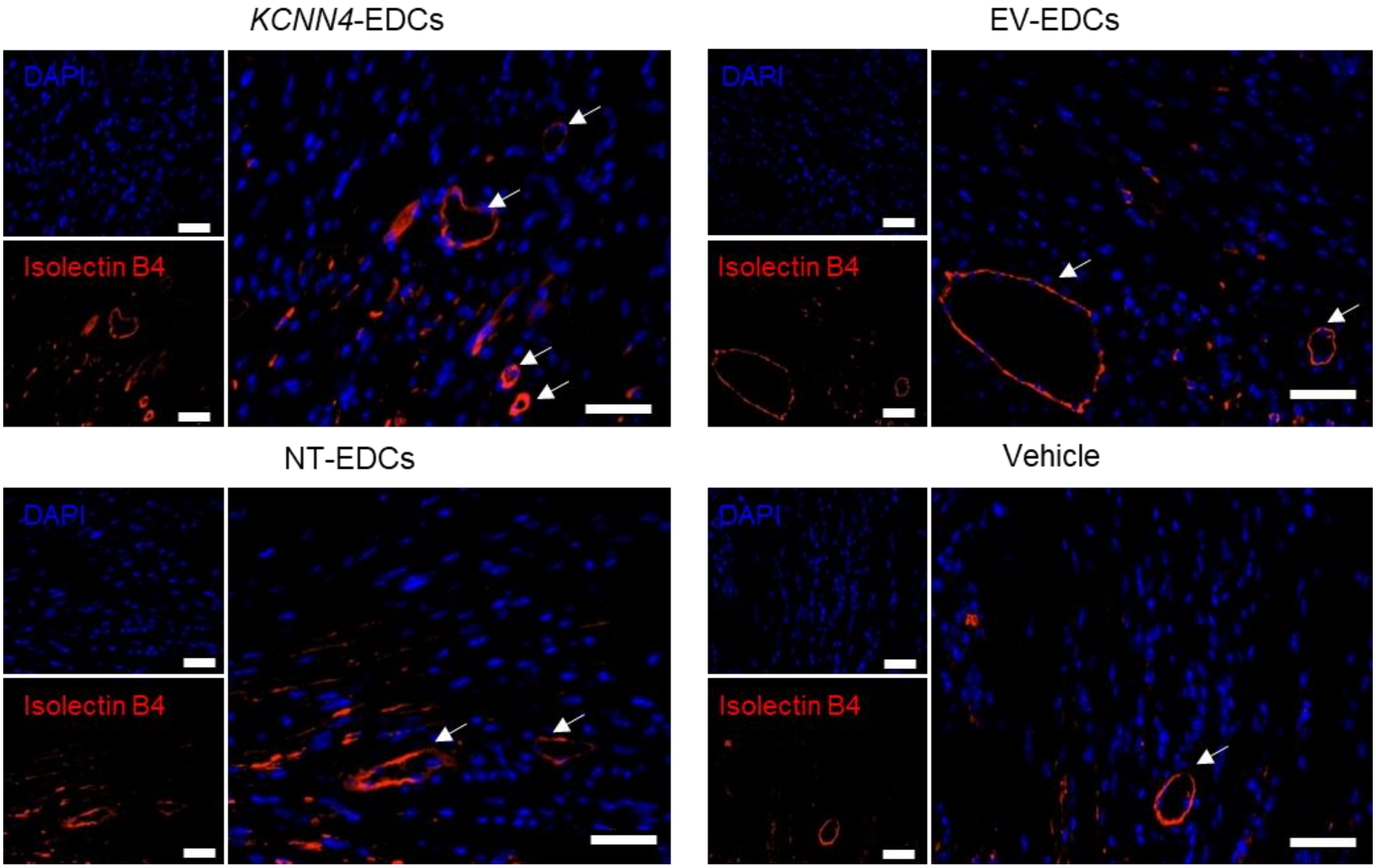

Supplementary Fig. 9. Representative images demonstrating vessel density within the peri-infarct region 4 weeks after LCA ligation. Vessels are denoted with a white arrow. Scale bar, 50 µm. The experiment was independently repeated in separate biological replicates 5 times for each cell type with similar results.

a

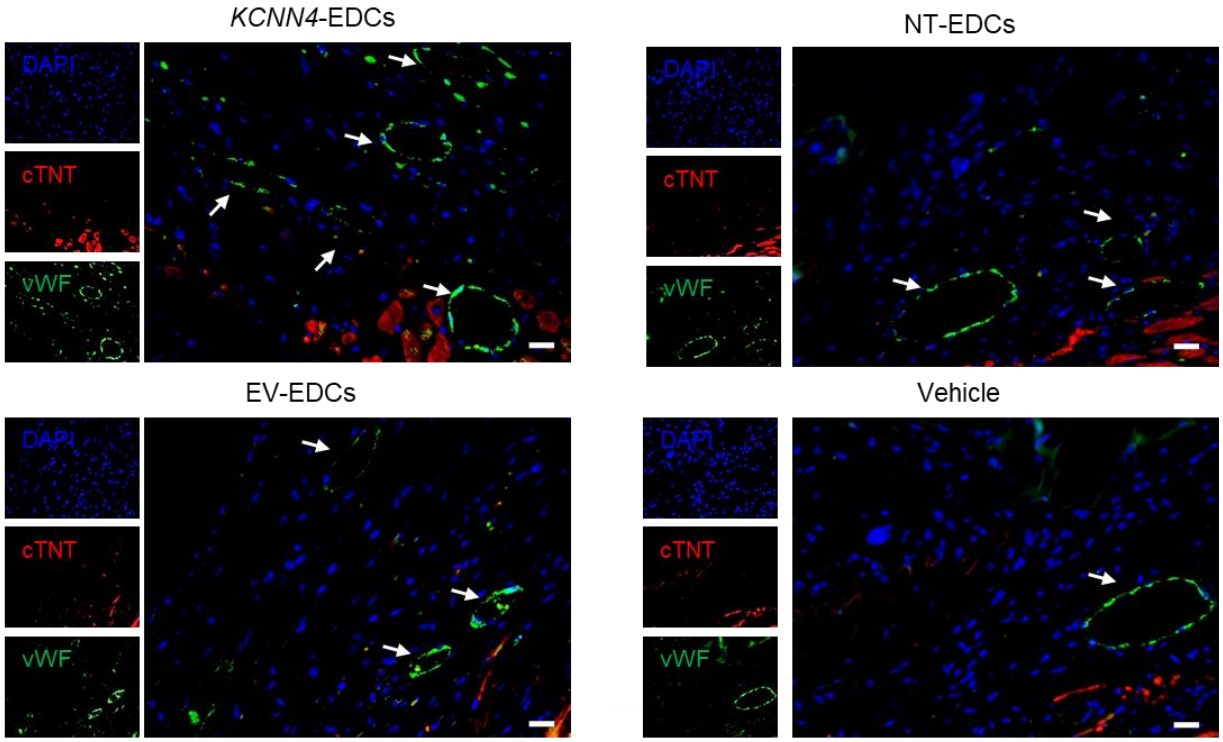

b

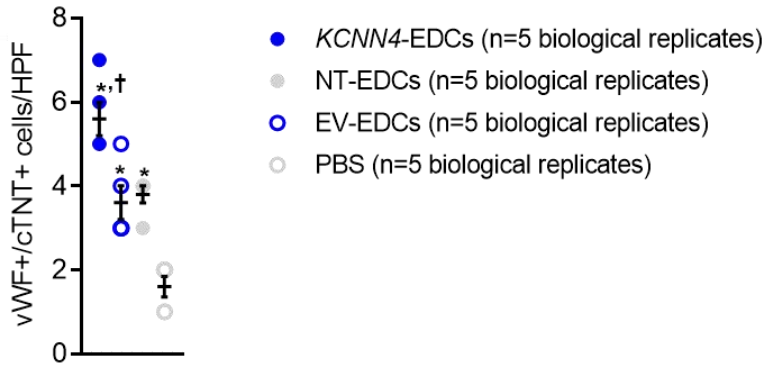

Supplementary Fig. 10. Effect of KCNN4 over-expression on peri-infarct vascularization. **a.** Representative images demonstrating vWF+/cTNT+ vessel density within the peri-infarct region 4 weeks after LCA ligation. Vessels are denoted with a white arrow. Scale bar, 50 µm. **b.** Aggregate data showing the effect of KCNN4 over expression on the number of vWF+/cTNT+/DAPI+ cells within the peri-infarct region 4 weeks after LCA ligation (n=5 biological repeats). *$P < 0.05$ vs. vehicle, †$P < 0.05$ vs. NT-EDCs or EV-EDCs. Data in **b** are shown as individual points, along with mean and SEM. For precise P values, see Supplemental Excel file. All data was analyzed using a one-way ANOVA with individual-mean comparisons by Bonferroni multiple comparisons test.

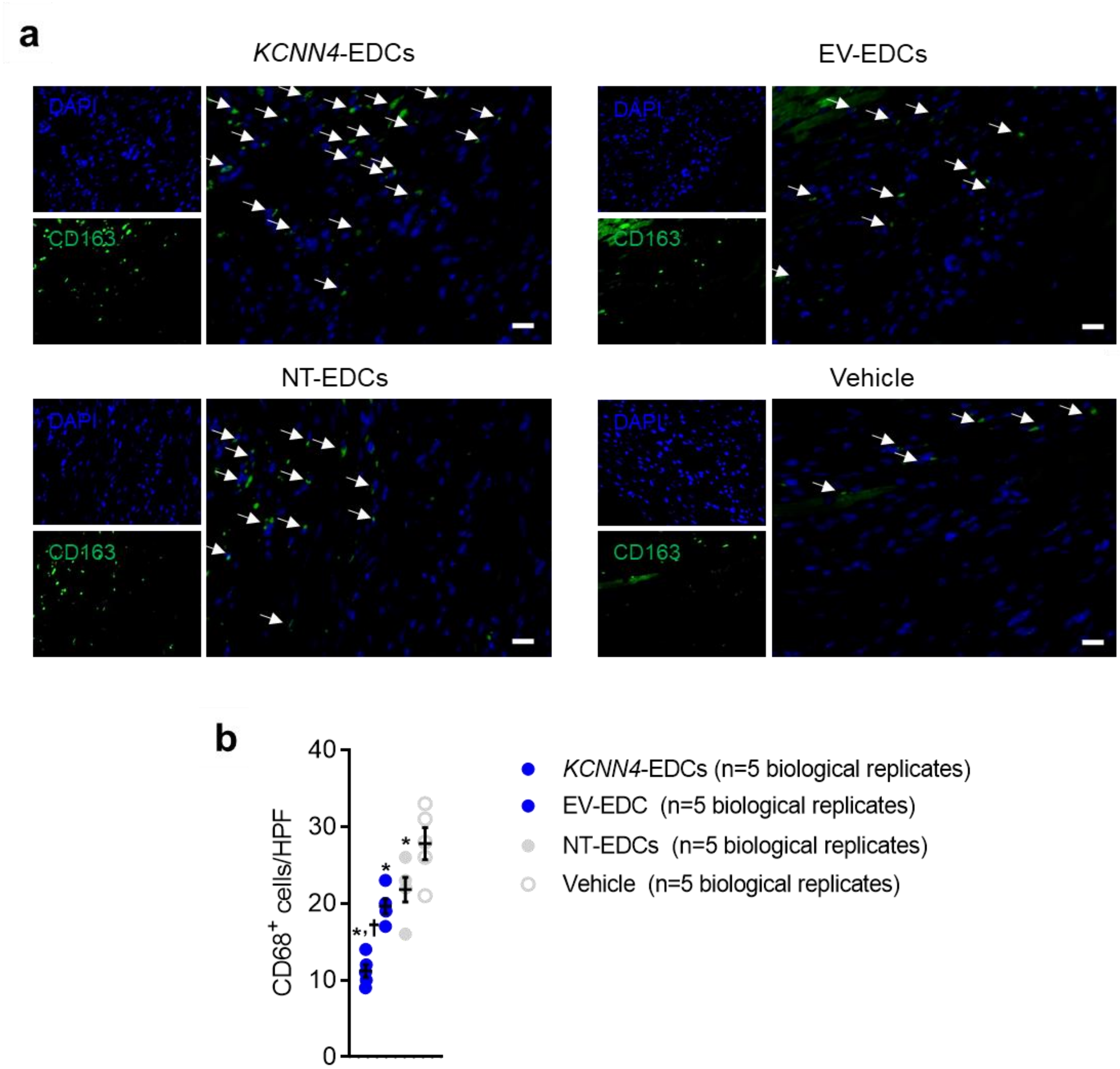

Supplementary Fig. 11. Effect of KCNN4 over-expression on pro-healing M2 macrophages. **a.** Representative images demonstrating CD163+/DAPI+ cells within the peri-infarct region 4 weeks after LCA ligation. Scale bar, 50 um. Arrows point to CD163+ cells. **b.** Aggregate data showing the effect of KCNN4 over expression on the number of CD163+/DAPI+ cells within the peri-infarct region 4 weeks after LCA ligation (n=5 biological repeats). Data in **b** are shown as individual points, along with mean and SEM. * $P < 0.05$ vs. vehicle, † $P < 0.05$ vs. NT-EDCs or EV-EDCs. For precise P values, see Supplemental Excel file. All data was analyzed using a one-way ANOVA with individual-mean comparisons by Bonferroni multiple comparisons test.

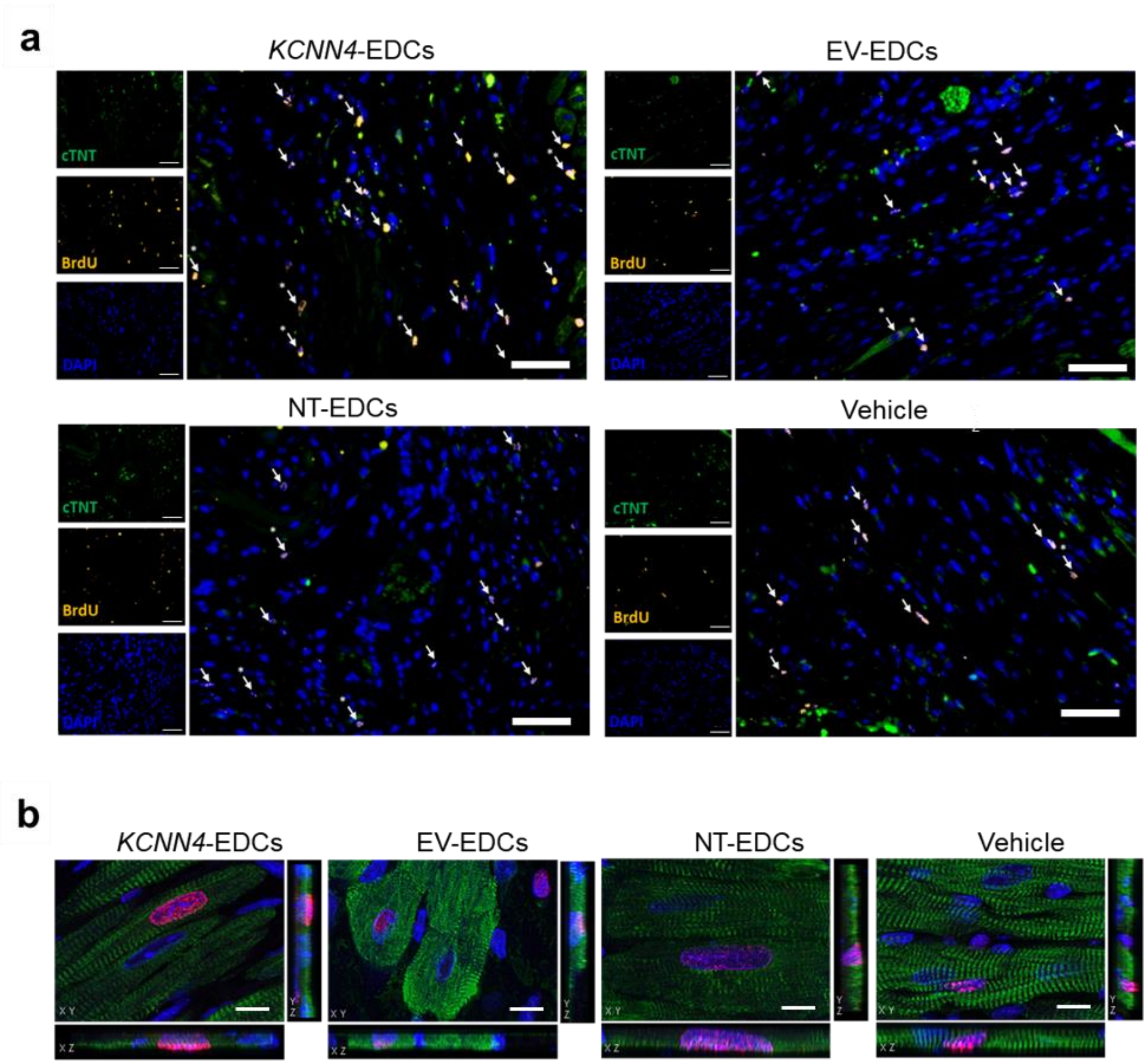

Supplementary Fig. 12. Representative images demonstrating BrdU$^+$/DAPI$^+$ cells within the peri-infarct region 4 weeks after LCA ligation. **a.** Cells positive for BrdU (BrdU$^+$, suggesting active proliferation) are denoted with a white arrow. Scale bar, 50 µm. The experiment was independently repeated in separate biological replicates 5 times for each cell type with similar results. **b.** Representative z-stacks images of BrdU$^+$ cells demonstrating nuclear localization. The experiment was independently repeated in separate biological replicates a total of 3 times for each cell type with similar results. Scale bar, 10 µm.

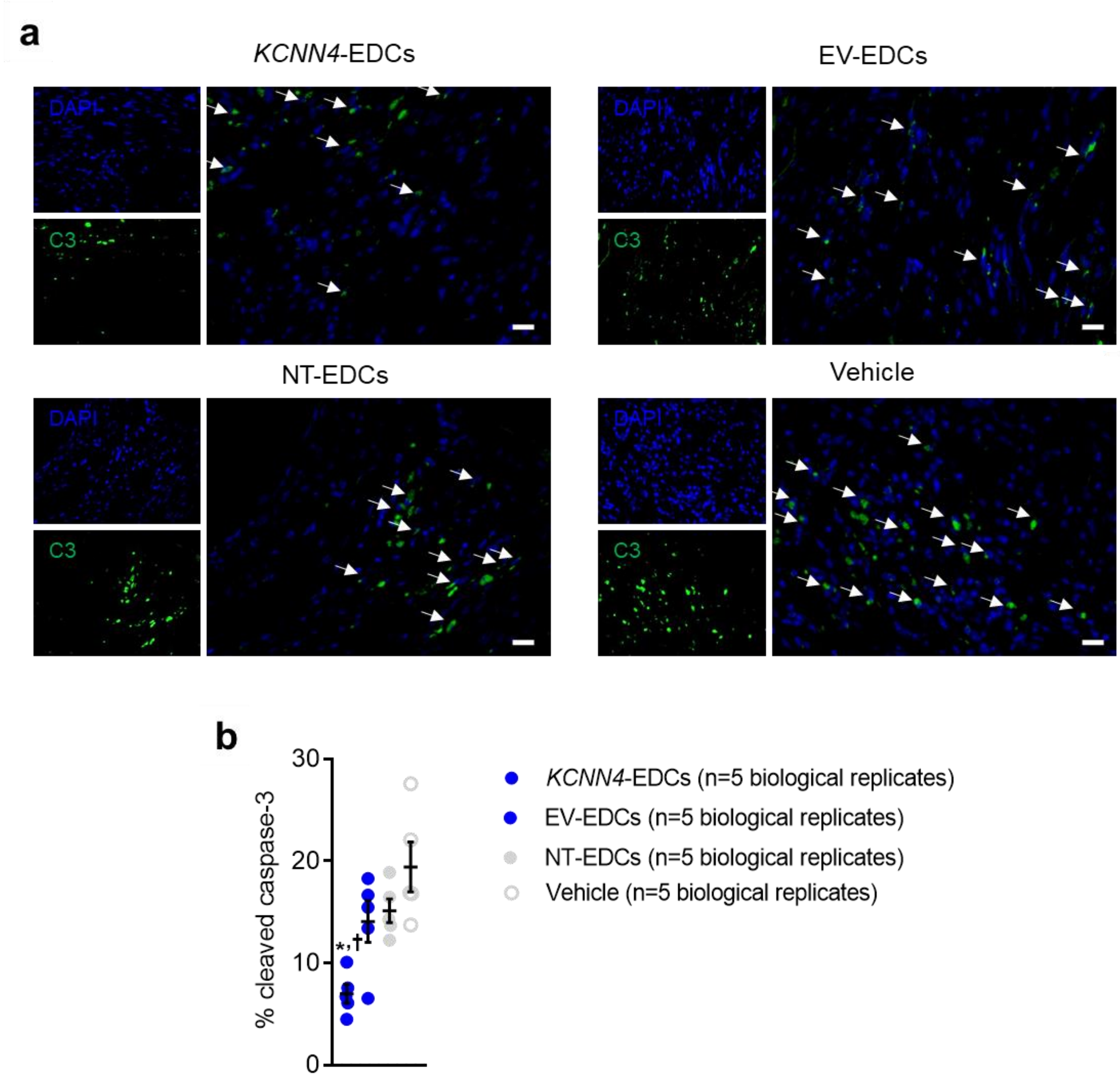

Supplementary Fig. 13. Effect of KCNN4 over-expression on markers of apoptosis. **a.** Representative images demonstrating cleaved caspase 3+/DAPI+ cells within the peri-infarct region 4 weeks after LCA ligation. Scale bar, 50 um. Arrows point to cleaved caspase 3+ cells. **b.** Aggregate data showing the effect of KCNN4 over expression on the number of cleaved caspase 3+/DAPI+ cells within the peri-infarct region 4 weeks after LCA ligation (n=5 biological repeats). Data in **b** are shown as individual points, along with mean and SEM. *P < 0.05 vs. vehicle, †P < 0.05 vs. NT-EDCs or EV-EDCs. For precise P values, see Supplemental Excel file. All data was analyzed using a one-way ANOVA with individual-mean comparisons by Bonferroni multiple comparisons test.

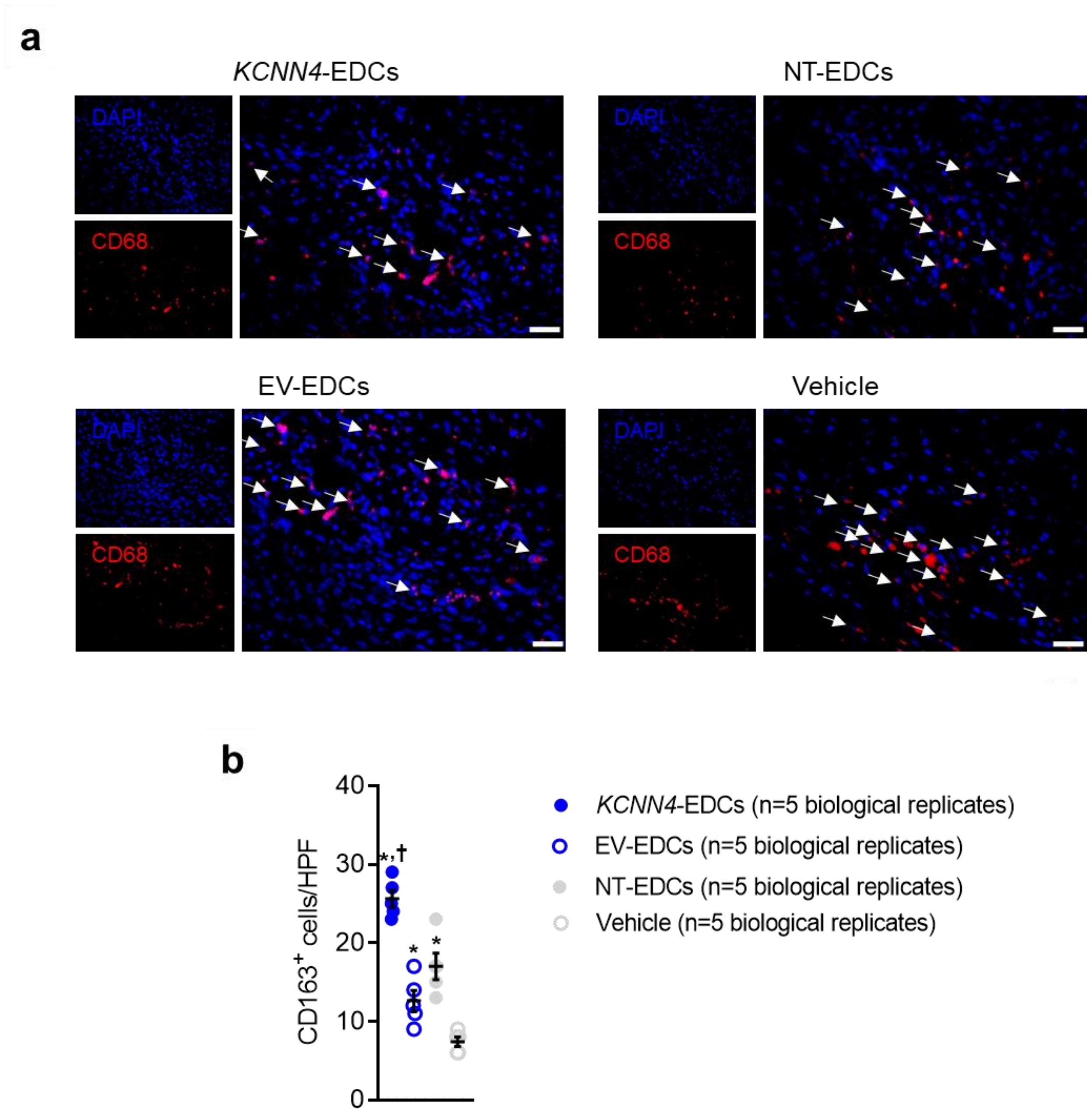

Supplementary Fig. 14. Effect of KCNN4 over-expression on cardiac macrophage numbers. **a.** Representative images demonstrating CD68+/DAPI+ cells within the peri-infarct region 4 weeks after LCA ligation. Scale bar, 50 um. Arrows point to CD68+ cells. **b.** Aggregate data showing the effect of KCNN4 over expression on the number of CD68+/DAPI+ cells within the peri-infarct region 4 weeks after LCA ligation (n=5 biological repeats). *$P < 0.05$ vs. vehicle, †$P < 0.05$ vs. NT-EDCs or EV-EDCs. Data in **b** are shown as individual points, along with mean and SEM. For precise P values, see Supplemental Excel file. All data was analyzed using a one-way ANOVA with individual-mean comparisons by Bonferroni multiple comparisons test.

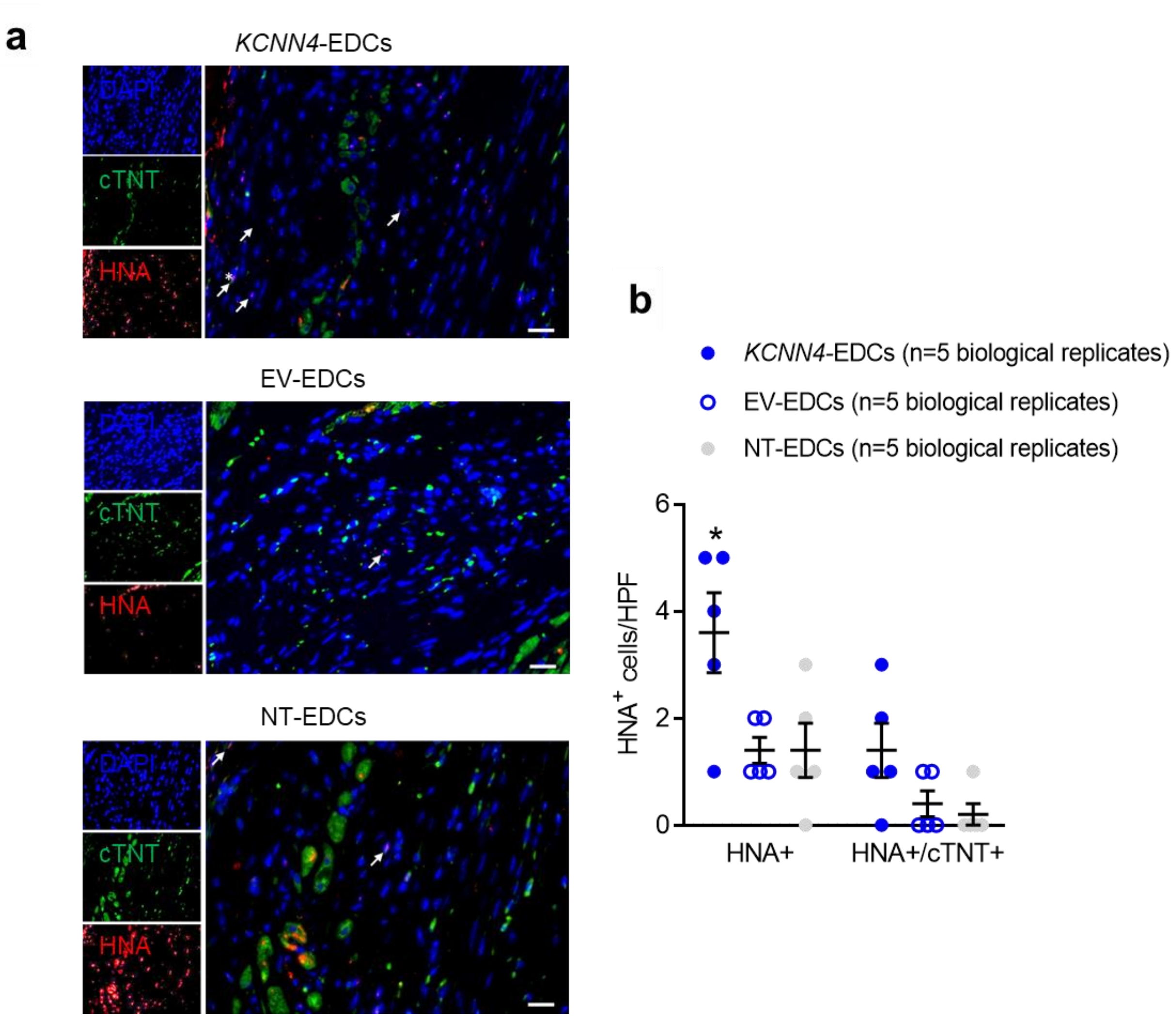

Supplementary Fig. 15. Effect of KCNN4 over-expression on EDC engraftment. **a.** Representative images demonstrating HNA+/cTNT+/DAPI+ cells within the peri-infarct region 4 weeks after LCA ligation. Scale bar, 50 um. Arrows point to HNA positive cells, star points to double HNA and cTNT positive cell. **b.** Aggregate data showing the effect of KCNN4 over-expression on the number of HNA+/DAPI+ or HNA+/cTNT+/DAPI+ cells within the peri-infarct region 4 weeks after LCA ligation (n=5 biological repeats). Data in **b** are shown as individual points, along with mean and SEM. **P* < 0.05 vs. NT-EDCs or EV-EDCs. For precise P values, see Supplemental Excel file. All data was analyzed using a one-way ANOVA with individual-mean comparisons by Bonferroni multiple comparisons test.

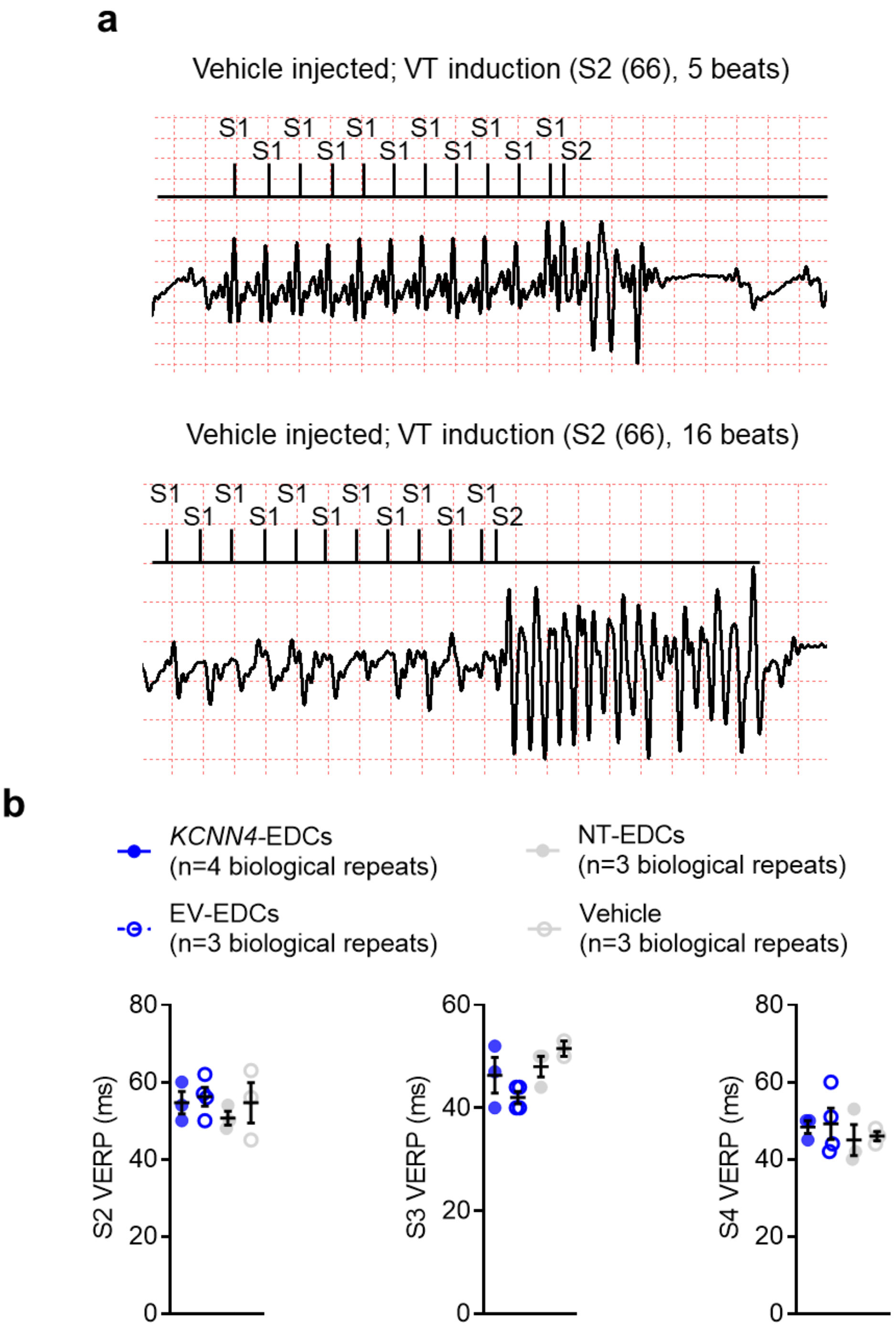

Supplementary Fig. 16. Electrophysiological effects of EDC or vehicle treatment on mice. **a.** Telemetry demonstrating induction of ventricular tachycardia in mice treated with vehicle. **b.** Effect of EDC or vehicle treatment on ventricular refractoriness. Data in **b** are shown as individual points, along with mean and SEM. One-way ANOVA with individual-mean comparisons by Bonferroni multiple comparisons test. N=4, 3, 3, 3 independent biological replicates for *KCNN4*-transfected EDCs (explant-derived cells), non-transduced (NT), empty-vector (EV) and vehicle-treated EDCs respectively.

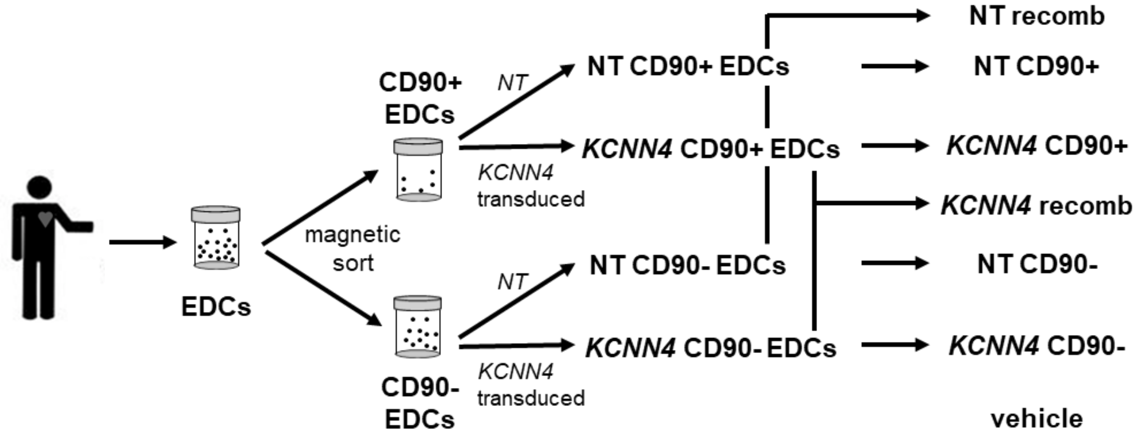

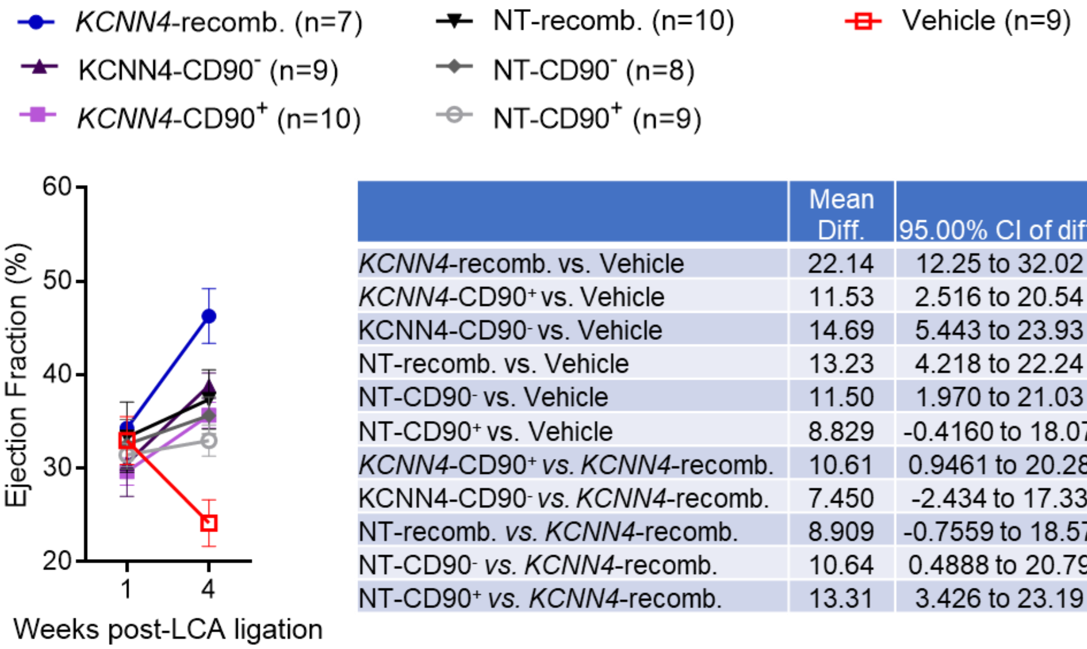

	Mean Diff.	95.00% CI of diff.	Adjusted P Value
KCNN4-recomb. vs. Vehicle	22.14	12.25 to 32.02	<0.001
KCNN4-CD90^{+} vs. Vehicle	11.53	2.516 to 20.54	0.004
KCNN4-CD90^{-} vs. Vehicle	14.69	5.443 to 23.93	<0.001
NT-recomb. vs. Vehicle	13.23	4.218 to 22.24	<0.001
NT-CD90^{-} vs. Vehicle	11.50	1.970 to 21.03	0.008
NT-CD90^{+} vs. Vehicle	8.829	-0.4160 to 18.07	0.07
KCNN4-CD90^{+} *vs. KCNN4*-recomb.	10.61	0.9461 to 20.28	0.02
KCNN4-CD90^{-} *vs. KCNN4*-recomb.	7.450	-2.434 to 17.33	0.28
NT-recomb. *vs. KCNN4*-recomb.	8.909	-0.7559 to 18.57	0.09
NT-CD90^{-} *vs. KCNN4*-recomb.	10.64	0.4888 to 20.79	0.03
NT-CD90^{+} *vs. KCNN4*-recomb.	13.31	3.426 to 23.19	0.002

Supplementary Fig. 17. In vivo study exploring the impact of KCNN4 over-expression on EDC mediated repair. The upper panel illustrates cell culture, magnetic separation, KCNN4 over-expression and the groups profiled. Lower panel outlines the impact of intramyocardial cell or vehicle injection 4 weeks after LCA ligation on echocardiographic ejection fraction. Data are shown as mean±SEM. One-way ANOVA with individual-mean comparisons by Bonferroni multiple comparisons test.

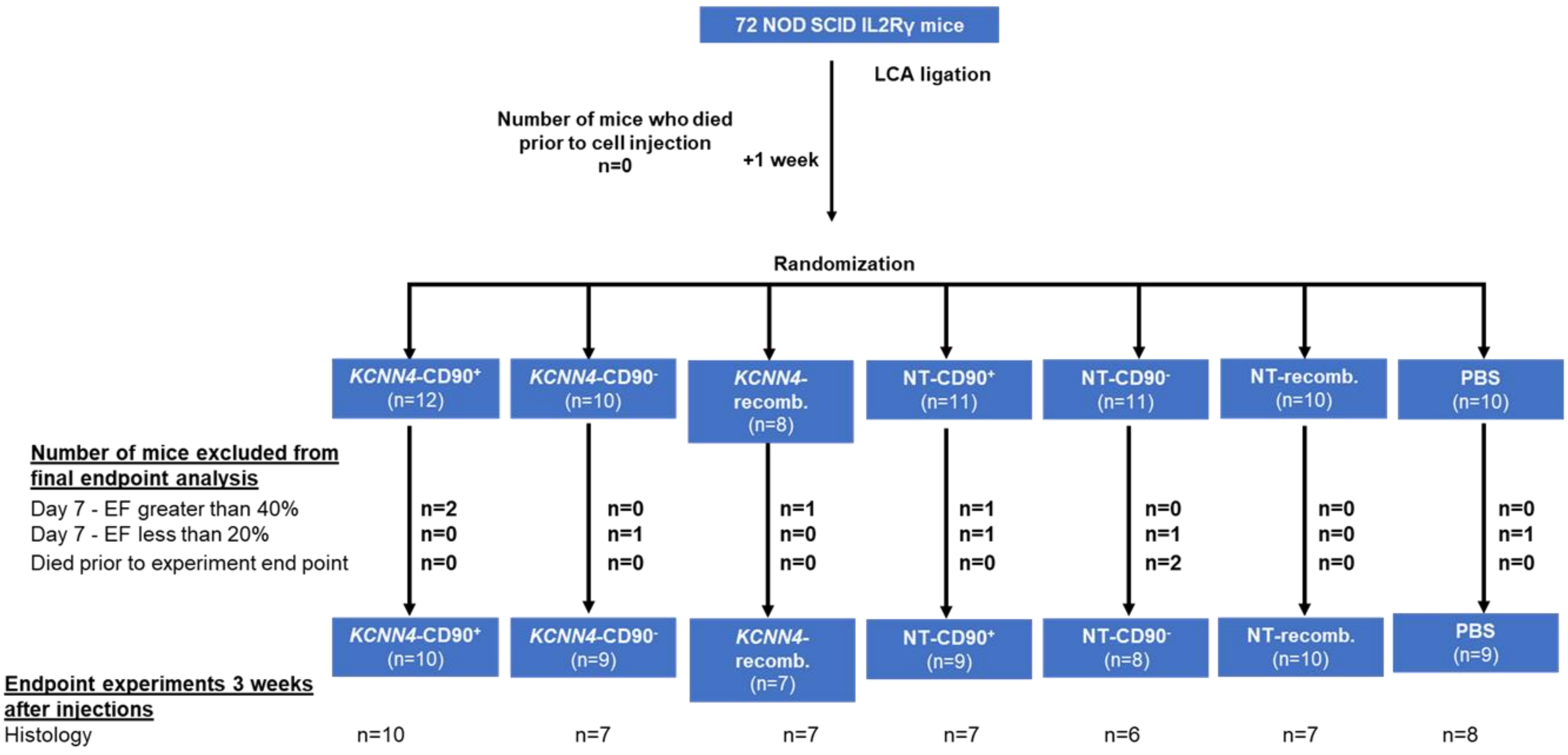

Supplementary Fig. 18. Group allocation, outcomes and endpoints for in vivo recombination cell product study.

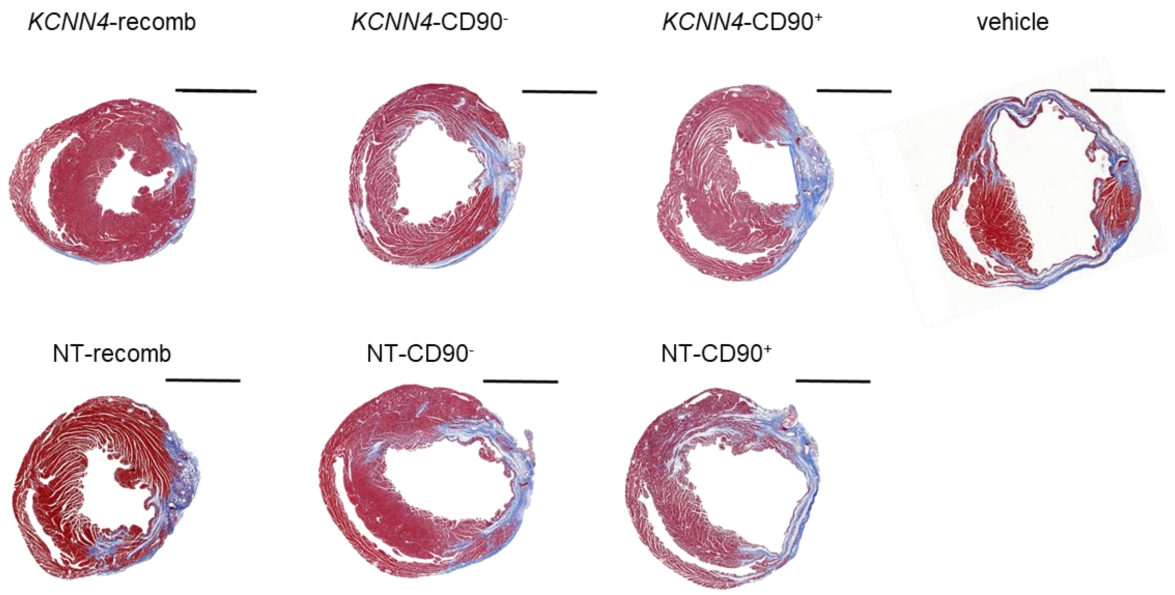

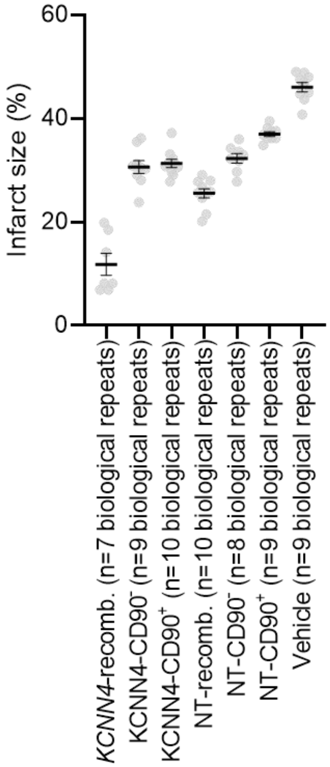

	Mean Diff.	95.00% CI of diff.	Adjusted P Value
KCNN4-recomb. vs. Vehicle	-34.21	-43.63 to -24.80	<0.001
KCNN4-CD90$^+$ vs. Vehicle	-14.56	-23.14 to -5.977	<0.001
KCNN4-CD90$^-$ vs. Vehicle	-15.35	-24.15 to -6.543	<0.001
NT-recomb. vs. Vehicle	-20.35	-28.94 to -11.77	<0.001
NT-CD90$^-$ vs. Vehicle	-12.72	-21.80 to -3.643	0.001
NT-CD90$^+$ vs. Vehicle	-8.870	-17.68 to -0.06362	0.047
KCNN4-CD90$^+$ *vs. KCNN4*-recomb.	-19.65	-28.86 to -10.45	<0.001
KCNN4-CD90$^-$ *vs. KCNN4*-recomb.	-18.86	-28.28 to -9.450	<0.001
NT-recomb. *vs. KCNN4*-recomb.	-13.86	-23.06 to -4.653	0.001
NT-CD90$^-$ *vs. KCNN4*-recomb.	-21.49	-31.16 to -11.83	<0.001
NT-CD90$^+$ *vs. KCNN4*-recomb.	-25.34	-34.76 to -15.93	<0.001

Supplementary Fig. 19. In vivo study exploring the impact of KCNN4 over-expression on EDC mediated repair. Representative images showing the effect of KCNN4 expression on Masson Trichrome stained sections. Scale bar, 2000 um. Quantitative data showing the effect of KCNN4 expression on scar size. Data are shown as individual points, along with mean and SEM. One-way ANOVA with individual-mean comparisons by Bonferroni multiple comparisons test.

a

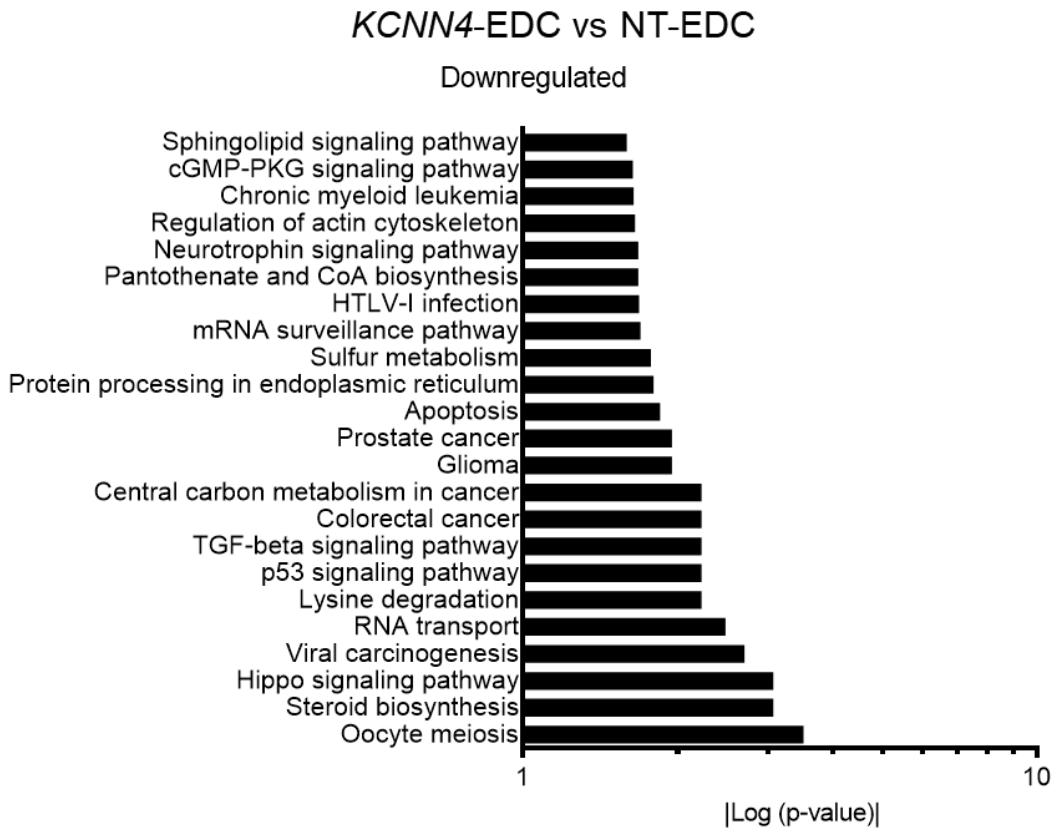
KCNN4-EDC vs NT-EDC
Downregulated
Sphingolipid signaling pathway
cGMP-PKG signaling pathway
Chronic myeloid leukemia
Regulation of actin cytoskeleton
Neurotrophin signaling pathway
Pantothenate and CoA biosynthesis
HTLV-I infection
mRNA surveillance pathway
Sulfur metabolism
Protein processing in endoplasmic reticulum
Apoptosis
Prostate cancer
Glioma
Central carbon metabolism in cancer
Colorectal cancer
TGF-beta signaling pathway
p53 signaling pathway
Lysine degradation
RNA transport
Viral carcinogenesis
Hippo signaling pathway
Steroid biosynthesis
Oocyte meiosis
1
10
|Log (p-value)|

b

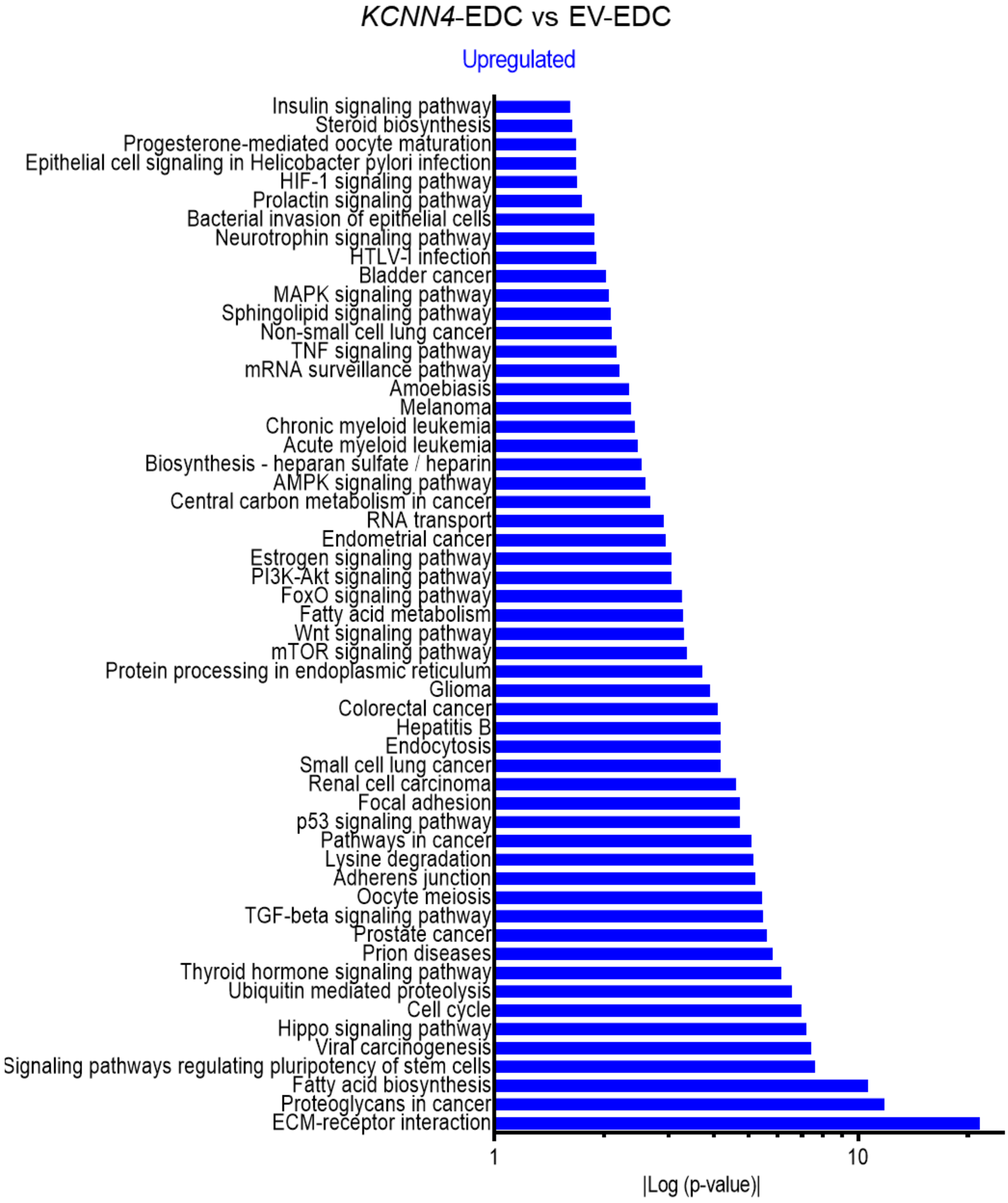

KCNN4-EDC vs EV-EDC
Upregulated
Insulin signaling pathway
Steroid biosynthesis
Progesterone-mediated oocyte maturation
Epithelial cell signaling in Helicobacter pylori infection
HIF-1 signaling pathway
Prolactin signaling pathway
Bacterial invasion of epithelial cells
Neurotrophin signaling pathway
HTLV-I infection
Bladder cancer
MAPK signaling pathway
Sphingolipid signaling pathway
Non-small cell lung cancer
TNF signaling pathway
mRNA surveillance pathway
Amoebiasis
Melanoma
Chronic myeloid leukemia
Acute myeloid leukemia
Biosynthesis - heparan sulfate / heparin
AMPK signaling pathway
Central carbon metabolism in cancer
RNA transport
Endometrial cancer
Estrogen signaling pathway
PI3K-Akt signaling pathway
FoxO signaling pathway
Fatty acid metabolism
Wnt signaling pathway
mTOR signaling pathway
Protein processing in endoplasmic reticulum
Glioma
Colorectal cancer
Hepatitis B
Endocytosis
Small cell lung cancer
Renal cell carcinoma
Focal adhesion
p53 signaling pathway
Pathways in cancer
Lysine degradation
Adherens junction
Oocyte meiosis
TGF-beta signaling pathway
Prostate cancer
Prion diseases
Thyroid hormone signaling pathway
Ubiquitin mediated proteolysis
Cell cycle
Hippo signaling pathway
Viral carcinogenesis
Signaling pathways regulating pluripotency of stem cells
Fatty acid biosynthesis
Proteoglycans in cancer
ECM-receptor interaction
1
10
|Log (p-value)|

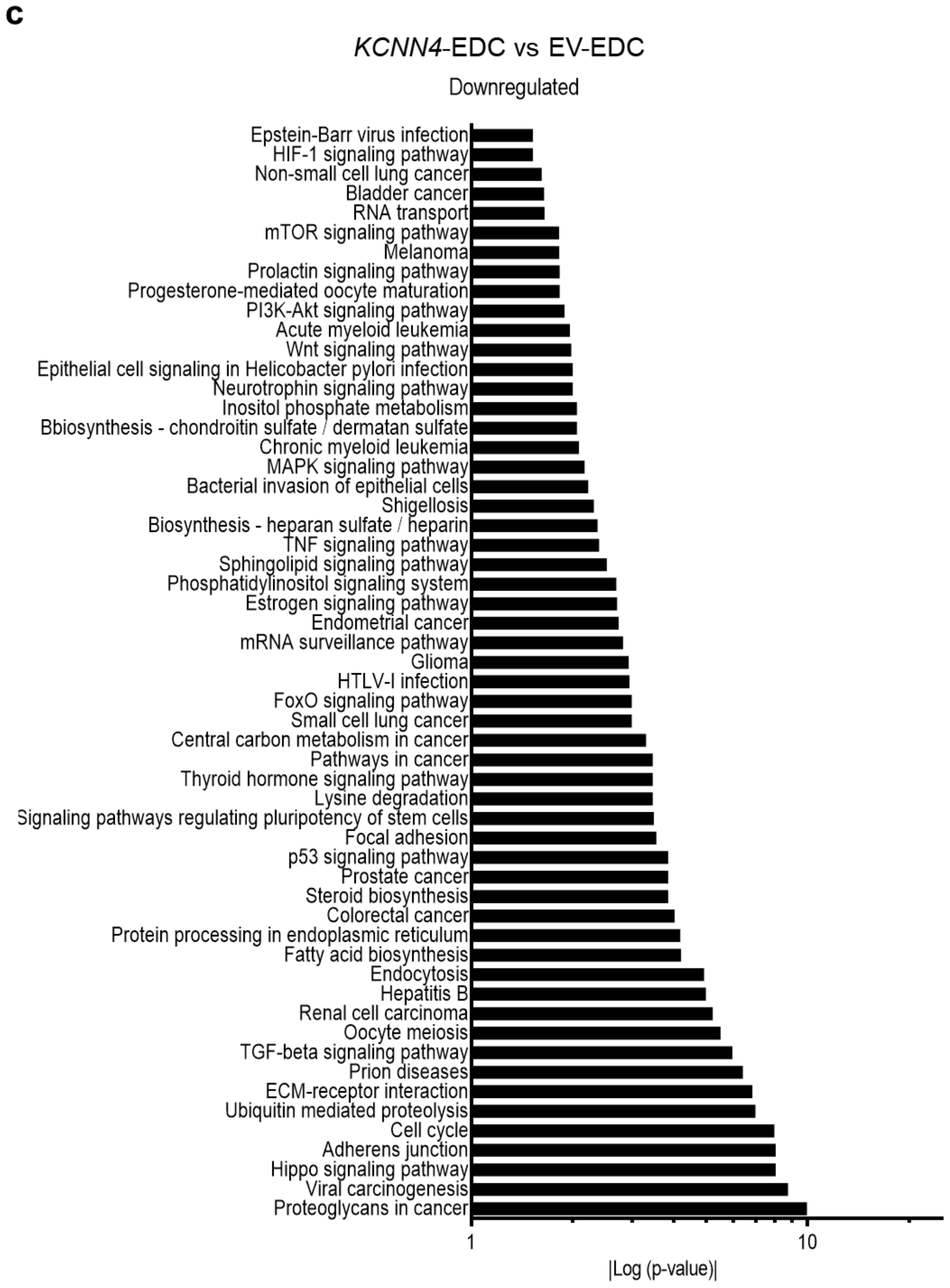

Supplementary Fig. 20. KEGG pathway analysis for molecular pathways predicted to be affected by miRNAs altered by *KCNN4* overexpression. Pathways associated with up- and down-regulated miRNAs in extracellular vesicles isolated from *KCNN4*-EDCs compared to **a.** NT-EDCs or **b, c.** EV-EDCs. For the comparison with NT-EDCs, only downregulated pathways were observed (as shown in **a**. For the comparison with EV-EDCs, both upregulated **b.** and downregulated **c.** pathways were noted. Online DIANA-TarBase tools were utilized to predict miRNA pathways based on experimentally validated miRNA interactions (DIANA-miRPath v3.0). Blue represents upregulated pathways, black downregulated. No pathways were upregulated in *KCNN4*-EDCs compared to NT-EDCs.

SUPPLEMENTARY TABLES

Supplementary Table 1. Clinical characteristics of atrial appendage donors used to obtain EDCs.

	Atrial Appendage donors *in vitro* study (n=8)	Atrial Appendage donors *in vivo* study (n=4)	*P* value
Age (years)	65±3	68±4	0.73
Body Mass Index (kg/m^2)	30±3	33±3	0.58
Gender (%male)	75%	50%	0.54
Diabetes	25%	0%	0.51
Hypertension	100%	50%	0.09
Dyslipidemia	63%	50%	1.00
Ongoing smoking	25%	50%	0.54
Thyroid disease	25%	25%	1.00
Peripheral vascular disease	13%	25%	1.00
Coronary artery disease	100%	75%	0.33
History of Myocardial Infarction	38%	25%	1.00
Valvular heart disease	50%	50%	1.00
Congestive heart failure	0%	25%	0.33
NYHA class	1.25±0.3	2.7±0.9	0.13
Left ventricular ejection fraction (%)	53±7	47±3	0.52
CCS class	1.8±0.7	3±0.1	0.37
Creatinine (µmol/L)	82±8	76±8	0.65
Hemoglobin A1c	5.8±0.2	5.8±0.3	1.00
Medications:			
Anti-platelet therapy	100%	100%	1.00
Beta-blocker	88%	50%	0.23
Statins	88%	25%	0.07
ACEI or ARB	88%	50%	0.23

NYHA = New York Heart Association; CCS = Canadian Cardiovascular Society; ACEI = Angiotensin-converting enzyme inhibitor; ARB = Angiotensin receptor blocker. P-values are based on 2-sided non-paired t-test (quantitative data) or Fisher's exact test (frequency data).

Supplementary Table 2. Echocardiographic measures of left ventricular function.

		LVEDV (μL)	LVESV(μL)	Stroke Volume (μL)	Ejection Fraction (%)	Fractional Area Change (%)	Cardiac Output (mL/min)
1 week post-LCA ligation	*KCNN4*-EDCs (n=12)	66.6±1.8	44.2±1.2	22.4±0.8	33.6±0.7	20.0±0.5	9.0±0.9
	NT-EDCs (n=13)	76.8±4.9	51.4±3.5	25.4±1.6	33.3±1.0	20.1±0.6	9.8±1.0
	EV-EDCs (n=12)	73.9±6.7	50.8±4.6	23.1±2.3	31.3±1.1	18.4±0.7	8.6±0.9
	Vehicle (n=14)	69.5±4.7	46.9±3.4	22.6±1.4	32.8±0.6	20.3±0.6	8.2±0.6
4 weeks post-LCA ligation	*KCNN4*-EDCs (n=12)	73.8±3.8	42.9±3.1	30.9±1.3[‡]	42.7±2.2[*,†,‡]	26.8±1.6[†,‡]	11.2±0.4[‡]
	NT-EDCs (n=13)	95.6±8.3	60.1±5.6	35.5±2.9[‡]	37.6±0.9[‡]	23.4±0.6[‡]	12.4±0.9[‡]
	EV-EDCs (n=12)	93.7±8.4	59.9±6.1	33.7±2.5[‡]	36.6±1.0[‡]	22.4±0.5[‡]	11.9±0.6[‡]
	Vehicle (n=14)	76.0±4.4	55.4±3.4	20.7±1.2	27.3±0.6	17.4±0.8	7.3±0.5

EDC = explant-derived cell; EV = empty vector; LCA = left coronary artery; LVEDV = left ventricular end diastolic volume; LVESV = left ventricular end systolic volume; NT = non treated.

* $P < 0.05$ vs. EV-EDCs, † $P < 0.05$ vs. NT-EDCs, ‡ $P < 0.05$ vs. vehicle. Statistics are by one-way ANOVA followed by Bonferroni multiple comparisons test.

Supplementary Table 3. Invasive hemodynamic measures of left ventricular function.

	Pmin (mmHg)	Pmean (mmHg)	Pdev (mmHg)	Pes (mmHg)	Ped (mmHg)	HR (bpm)	Ea (mmHg/uL)	dP/dt max (mmHg/s)
KCNN4-EDCs (n=11)	4.0±0.6[‡]	39.2±1.9[‡]	84.2±2.4[*,†,‡]	82.5±2.6[*,‡]	9.5±0.9[‡]	557±8	5.3±0.3	7934±198[*,†,‡]
NT-EDCs (n=13)	2.5±0.6	34.3±1.4[‡]	75.4±2.0[‡]	71.3±3.0	7.3±0.9	557±11	4.5±0.3	6542±356[‡]
EV-EDCs (n=10)	3.8±0.7	36.1±2.1[‡]	75.2±1.1[‡]	76.6±1.8[‡]	7.6±1.2	575±9	5.2±0.4	6518±196[‡]
Vehicle (n=10)	1.6±0.3	24.8±1.3	66.5±1.2	65.0±1.2	4.0±0.5	561±11	5.5±0.7	5674±166

	dP/dt min (mmHg/s)	dV/dt max (uL/s)	dV/dt min (uL/s)	P@dV/dt max (mmHg)	P@dP/dt max (mmHg)	V@dP/dt max (uL)	V@dP/dt min (uL)
KCNN4-EDCs (n=11)	-6629±282[*,†,‡]	758±40	-796±52	12.9±3.1	54.3±1.7[*,‡]	32.5±3.9	18.0±3.2
NT-EDCs (n=13)	-5437±369	766±85	-893±68	12.9±5.3	45.1 ±2.2[‡]	33.1±4.0	18.8±3.8
EV-EDCs (n=10)	-5443±144	745±64	-846±95	12.7±2.9	47.9±2.0[‡]	36.8±3.6	23.3±3.2
Vehicle (=10)	-4816±107	745±146	-826±151	8.7±3.4	36.1±0.8	34.1±3.5	22.8±2.7

	SW (mmHg*uL)	CO (uL/min)	SV (uL)	Vmax (uL)	Vmin (uL)	Ves (uL)	Ved (uL)	Pmax (mmHg)
KCNN4-EDCs (n=11)	1144±79[‡]	9472±556	16.6±1.0	35.6±3.8	17.2±3.2	19.2±3.3	31.5±3.5	88.2±2.2[*,†,‡]
NT-EDCs (n=13)	997±76	9445±692	17.0±1.2	35.0±3.9	18.0±3.7	19.6±3.8	32.8±4.0	77.8±1.9[‡]
EV-EDCs (n=10)	981±113	9473±908	16.6±1.7	34.4±4.1	22.3±3.1	24.8±3.3	37.0±3.6	80.7±1.6[‡]
Vehicle (n=10)	713±92	8033±1024	14.4±1.9	36.4±3.5	22.1±2.7	24.6±2.7	33.9±2.9	68.0±1.3

	PVA (mmHg/uL)	PE (mmHg/uL)	CE
KCNN4-EDCs (n=13)	2092±213	949±183	0.8±0.2
NT-EDCs (n=13)	1980±271	789±162	0.6±0.1
EV-EDCs (n=10)	1757±229	801±198	0.6±0.1
Vehicle (=10)	1308±177	595±112	0.4±0.1

CE = cardiac efficiency; CO = cardiac output; dP/dtmax = maximum derivative of pressure; dP/dtmin = minimum derivative of pressure; dV/dtmax = maximum derivative of volume; dV/dtmin = minimum derivative of volume; Ea = arterial elastance; EDC = explant-derived cell; EV = empty vector; HR = heart rate; NT = non treated; P@dP/dtmax = pressure at maximum derivative of pressure;

P@dV/dtmax = pressure at maximum derivative of volume; Ped = end diastolic pressure; Pes = end systolic pressure; Pmax = maximum pressure; Pmean = mean pressure; Pmin = minimum pressure; PVA = pressure-volume area; PE = potential energy; SV = stroke volume; SW = stroke work; TAU = isovolumic relaxation constant; V@dP/dtmax = volume at maximum derivative of pressure; V@dP/dtmin = volume at minimum derivative of pressure; Ved = end diastolic volume; Ves = end systolic volume; Vmax = maximum volume; Vmin = minimum volume. * $P < 0.05$ vs. EV-EDCs, † $P < 0.05$ vs. NT-EDCs, ‡ $P < 0.05$ vs. vehicle. One-way ANOVA followed by Bonferroni multiple comparisons test.

Supplementary Table 4. Echocardiographic measures of left ventricular function examining the long-term durability of cell transplantation on heart function.

Weeks post LCA ligation		LVEDV (μL)	LVESV(μL)	Stroke Volume (μL)	Ejection Fraction (%)	Fractional Area Change (%)	Cardiac Output (mL/min)
Week 1	*KCNN4*-EDCs (n=7)	73.8±8.8	48.5±5.8	25.2±3.0	34.2±0.6	20.0±1.0	9.3±1.2
	NT-EDCs (n=7)	77.1±6.3	52.1±4.5	25.0±1.9	32.6±1.5	20.0±0.5	9.4±0.9
	EV-EDCs (n=10)	78.0±5.2	53.8±3.5	24.1±1.9	30.8±1.3	19.8±0.6	8.6±0.7
	Vehicle (n=7)	70.9±4.8	48.0±3.3	23.0±1.6	32.3±1.0	20.1±0.8	8.4±0.7
Week 4	*KCNN4*-EDCs (n=7)	62.6±5.0	33.4±3.0$^{†, *}$	29.2±2.2*	46.8±2.9$^{†, *}$	29.0±1.6$^{†, *}$	10.6±0.3
	NT-EDCs (n=7)	90.1±9.4	57.9±6.6	32.2±3.0*	36.2±1.3*	22.3±0.5*	11.5±0.6
	EV-EDCs (n=10)	73.5±6.7	47.1±4.4	26.4±2.9*	35.8±3.0*	21.7±0.6*	9.1±1.1
	Vehicle (n=7)	78.2±6.6	57.2±4.9	21.0±1.9	26.8±1.0	16.1±0.8	8.2±0.8
Week 8	*KCNN4*-EDCs (n=7)	65.1±9.3	33.9±6.9	31.2±2.7	49.6±2.2$^{†, *}$	31.1±3.4$^{†, *}$	11.6±1.1
	NT-EDCs (n=7)	80.5±2.0	48.5±7.6	32.0±4.4	39.9±1.1*	24.4±1.2*	11.7±1.4
	EV-EDCs (n=10)	69.9±4.1	42.7±7.6	27.2±4.4	38.8±1.8*	22.9±1.2*	11.6±1.4
	Vehicle (n=7)	80.7±5.9	61.5±2.2	19.2±3.7	23.9±1.6	14.5±0.1	7.0±1.4

EDC = explant-derived cell; LVEDV = left ventricular end diastolic volume; LVESV = left ventricular end systolic volume; NT = non treated. *$P < 0.05$ vs. vehicle treated mice, †$P < 0.05$ vs. EV- or NT-EDCs treated mice. One-way and two-way ANOVA with individual-mean comparisons by Bonferroni multiple comparisons test for each time point.

Supplementary Table 5. Echocardiographic measures of left ventricular function examining the effect of cell injection on normal heart function.

		LVEDV (μL)	LVESV(μL)	Stroke Volume (μL)	Ejection Fraction (%)	Fractional Area Change (%)	Cardiac Output (mL/min)
Day 0	*KCNN4*-EDCs (n=10)	65.0±2.8	24.1±1.0	40.9±2.4	62.6±1.8	18.3±1.8	17.2±1.4
	NT-EDCs (n=10)	59.8±2.2	21.6±1.1	38.2±1.5	63.9±1.0	15.9±1.0	16.4±1.1
Day 28	*KCNN4*-EDCs (n=10)	68.4±3.2	25.0±1.0	43.4±2.8	63.1±1.4	16.0±1.0	21.4±1.6
	NT-EDCs (n=10)	65.0±3.2	25.8±1.2	39.1±2.5	59.9±1.5	13.8±0.8	18.0±1.6

EDC = explant-derived cell; LVEDV = left ventricular end diastolic volume; LVESV = left ventricular end systolic volume; NT = non treated.

Supplementary Table 6. Effect of *KCNN4* overexpression on electrophysiological parameters.

	RR (ms)	PR (ms)	QRS (ms)	QT (ms)	QTc (ms)
KCNN4-EDCs	165±3	51±3	27±2	85±2	209±6
NT-EDCs	174±12	56±2	31±2	90±5	217±10
EV-EDCs	157±3	51±2	24±2	82±2	208±5
Vehicle	168±5	47±3	30±2	94±5	228±9

EDC = explant-derived cell; EV = empty vector; NT = non-transduced.

Supplementary Table 7. Echocardiographic measures of left ventricular function examining the impact of KCNN4 over-expression on EDC mediated repair.

		LVEDV (μL)	LVESV(μL)	Stroke Volume (μL)	Ejection Fraction (%)	Fractional Area Change (%)	Cardiac Output (mL/min)
1 week post LCA ligation	*KCNN4*-recomb. (n=7)	71.1±5.8	46.7±3.9	24.4±2.0	34.3±0.6	19.9±0.7	8.7±0.8
	KCNN4-CD90$^-$ (n=9)	69.0±5.7	48.0±4.3	20.9±1.6	30.3±3.5	17.9±0.7	7.9±0.6
	KCNN4-CD90$^+$ (10)	82.8±9.6	57.2±6.4	24.5±3.6	29.6±1.5	17.8±1.2	9.6±1.5
	NT-recomb. (n=10)	70.5±2.9	47.2±1.9	23.3±1.2	33.0±3.7	20.7±0.8	5.6±0.3
	NT-CD90$^-$ (n=8)	80.1±7.8	53.8±5.4	26.3±2.5	32.8±2.7	21.2±0.6	8.8±1.0
	NT-CD90$^+$ (n=9)	69.9±6.4	47.9±4.7	22.0±1.8	31.5±1.2	19.4±0.7	8.3±0.6
	PBS (n=9)	92.2±11.1	61.4±7.4	30.8±3.7	33.4±2.6	20.4±0.5	10.8±1.2
Day 28 post LCA ligation	*KCNN4*-recomb. (n=7)	77.3±7.6	42.6±5.8	34.6±1.9‡	46.5±3.0‡	29.2±2.6‡	12.2±0.7‡
	KCNN4-CD90$^-$ (n=9)	76.5±6.7	46.8±4.0	29.7±2.8‡	38.8±1.4‡	23.4±0.8*,‡	11.2±0.6‡
	KCNN4-CD90$^+$ (10)	72.2±9.5	46.1±5.6	26.1±3.9	35.7±1.4*,‡	21.0±1.0*,‡	9.2±1.5
	NT-recomb. (n=10)	82.5±7.6	51.6±4.7	30.9±3.0‡	37.3±3.2‡	22.9±0.9*,‡	10.8±0.9‡
	NT-CD90$^-$ (n=8)	98.5±5.0	63.7±1.0	34.8±4.0‡	35.6±2.0*,‡	23.1±1.7*,‡	11.9±0.7‡
	NT-CD90$^+$ (n=9)	76.7±5.9	51.4±4.2	25.3±1.8	33.1±1.7*	20.1±0.7*,‡	9.3±0.7
	PBS (n=9)	73.9±7.8	55.5±6.0	18.4±1.9	24.9±2.5	14.5±0.4	6.7±0.7

LVEDV = left ventricular end diastolic volume; LVESV = left ventricular end systolic volume; NT = non treated.
* $P < 0.05$ vs. *KCNN4*-recomb., ‡ $P < 0.05$ vs. vehicle.
One-way ANOVA with individual-mean comparisons by Bonferroni multiple comparisons test.

Supplementary Table 8. Top 10 miRNAs within adherent explant-derived cell extracellular vesicles involved with cardiomyocyte proliferation, salvage, and modulating cardiac fibrosis.

Name	Biological role
miR-199a	Promotes cardiomyocyte proliferation[1]
miR-93	Protects against ischemia-reperfusion injury[2]
miR-23a	Promotes cardiomyocyte proliferation[3]
miR-125b	Protects cardiomyocytes against p53 mediated apoptosis[4]
miR-199a+miR-199b	Promotes cardiomyocyte proliferation[5]
miR-21	Protects cardiomyocyte from oxidative stress[6]
miR-22	Regulates cardiac tissue fibrosis[7]
miR-495	Promotes cardiomyocyte proliferation[8]
miR-873	Inhibits RIPK1/RIPK3-mediated necrotic cell death in cardiomyocytes[9]
let-7b	Protects transplanted mesenchymal stem cells from apoptosis[10]

Supplementary Table 9. Effect of *KCNN4* overexpression on the miRNA profile within extracellular vesicles compared to non-transduced explant-derived cells.

Name	*P*-adjusted	*P*-value	Log2 (Fold change)
Downregulated miRNAs			
miR-1246	0.001392	1.02E-05	-6.02242
miR-4531	0.004192	9.25E-05	-5.0545
miR-548n	0.003996	5.88E-05	-5.26002
miR-603	0.006064	0.000178	-4.76565

Supplementary Table 10. Effect of *KCNN4* overexpression on the miRNA profile within extracellular vesicles compared to empty vector explant-derived cells.

Name	*P*-adjusted	*P*-value	Log2 (Fold change)
Upregulated miRNAs			
let-7a	7.18E-05	5.28E-07	7.117411
miR-100	0.044937	0.003896	2.640891
miR-199b	0.009134	0.000269	2.600605
miR-191	0.016288	0.000637	3.028148
miR-181a	0.005024	0.000111	3.033296
miR-21	0.040347	0.0027	2.603005
miR-22	0.016288	0.000719	2.630868
miR-25	0.023237	0.001196	2.517763
miR-15b	0.040347	0.002967	2.307322
miR-93	0.047076	0.005538	2.935141
miR-99a	0.036286	0.002134	2.370586
miR-15a	0.044937	0.004436	2.222103
miR-29b	0.044937	0.004626	2.417488
Downregulated miRNAs			
miR-144	0.044973	0.00496	-3.26627
miR-182	0.044937	0.003966	-3.51073
miR-451a	0.000382	5.61E-06	-5.79156

Supplementary Table 11. List of primers used.

Transcript	Primer Sequence
**ALU**	*Fw* 5'-CAT GGT GAA ACC CCG TCT CTA-3'<br>*Rv* 5'-GCC TCA GCC TCC CGA GTA G-3'<br>*Pr* 5'-/56-FAM/ATT AGC CGG/ZEN/GCG TGG TGG CG/3IABkFQ/-3'
**GAPDH**	*Fw* 5'-CAA CTG CTT AGC ACC CCT GG-3'<br>*Rv* 5'-GGC CAT CCA CAG TCT TCT GG-3'
**KCNN4**	*Fw* 5'- TAA AGC TTG GCC ACG AAC CA-3'<br>*Rv* 5'- TCC TGC TCA ACG CTT CCT AC-3'

Fw: Forward Primer
Rv: Reverse Primer
Pr: Probe

SUPPLEMENTARY REFERENCES

1. Eulalio, A., et al. Functional Screening Identifies Mirnas Inducing Cardiac Regeneration. *Nature* **492**, 376-381 (2012).

2. Ke, Z.P., Xu, P., Shi, Y., Gao, A.M. Microrna-93 Inhibits Ischemia-Reperfusion Induced Cardiomyocyte Apoptosis by Targeting Pten. *Oncotarget* **7**, 28796-28805 (2016).

3. Pandey, R., Yang, Y., Jackson, L., Ahmed, R.P. Micrornas Regulating Meis1 Expression and Inducing Cardiomyocyte Proliferation. *Cardiovasc. Regen Med* **3**, e1468 (2016).

4. Wang, X., et al. Microrna-125b Protects against Myocardial Ischaemia/Reperfusion Injury *Via* Targeting P53-Mediated Apoptotic Signalling and TRAF6. *Cardiovasc. Res.* **102**, 385-395 (2014).

5. Lesizza, P., Prosdocimo, G., Martinelli, V., Sinagra, G., Zacchigna, S., Giacca, M. Single-Dose Intracardiac Injection of Pro-Regenerative Micrornas Improves Cardiac Function after Myocardial Infarction. *Circ. Res.* **120**, 1298-1304 (2017).

6. Xiao, J., et al. Cardiac Progenitor Cell-Derived Exosomes Prevent Cardiomyocytes Apoptosis through Exosomal Mir-21 by Targeting PDCD4. *Cell Death Dis.* **7**, e2277 (2016).

7. Hong, Y., et al. Mir-22 May Suppress Fibrogenesis by Targeting TGFbetaR I in Cardiac Fibroblasts. *Cell. Physiol. Biochem.* **40**, 1345-1353 (2016).

8. Clark, A.L., Naya, F.J. MicroRNAs in the Myocyte Enhancer Factor 2 (MEF2)-regulated Gtl2-Dio3 Noncoding RNA Locus Promote Cardiomyocyte Proliferation by Targeting the Transcriptional Coactivator Cited2. *J. Biol. Chem.* **290**, 23162-23172 (2015).

9. Wang, K., et al. The Long Noncoding Rna NRF Regulates Programmed Necrosis and Myocardial Injury During Ischemia and Reperfusion by Targeting Mir-873. *Cell Death Differ.* **23**, 1394-1405 (2016).

10. Cheng, J., Zhang, P., Jiang, H. Let-7b-Mediated Pro-Survival of Transplanted Mesenchymal Stem Cells for Cardiac Regeneration. *Stem Cell Res. Ther.* **6**, 216 (2015).
